# Coupling Nanostructured Plasmon–Strain Microwave Waveguide to Spin Defects in Hexagonal Boron Nitride for High‐Sensitivity Quantum Sensors

**DOI:** 10.1002/adma.202516761

**Published:** 2026-03-28

**Authors:** Naveed Hussain, Sumukh Vaidya, Saakshi Dikshit, Shahriar Esmaeili, Paul Schmalenberg, Hayate Yamano, Katsunori Danno, Biswajit Sahoo, Shougo Higashi, Ercan M. Dede, Tongcang Li, Debasish Banerjee, Songtao Wu

**Affiliations:** ^1^ Toyota Research Institute of North America Ann Arbor Michigan USA; ^2^ Department of Physics and Astronomy Purdue University West Lafayette Indiana USA; ^3^ Elmore Family School of Electrical and Computer Engineering Purdue University West Lafayette Indiana USA; ^4^ Toyota Motor Corporation Toyota Technical Center Higashi‐Fuji Susono Shizuoka Japan; ^5^ Department of Physics University of California San Diego California USA

**Keywords:** boron vacancy, hexagonal boron nitride, optically detected magnetic resonance, plasmonic nanoresonators, quantum sensor, spin defects, strain

## Abstract

Despite seamless integration of hexagonal boron nitride (hBN) with on‐chip devices, the intrinsically low optical quantum yield of spin‐active boron vacancy ($V_{B}^{-}$) defects remains a significant limitation to the sensitivity of hBN‐based quantum sensors. Here, we demonstrate an hBN quantum sensor with enhanced quantum yield and high DC magnetic field sensitivity (η_DC_), achieved by coupling $V_{B}^{-}$ defects in hBN with a nanostructured plasmon‐strain microwave waveguide architecture.This platform is realized by fabricating arrays of alumina‐coated gold nanopillars, or plasmonic nanoresonators (PNRs), onto the constricted region of a microwave‐efficient, single‐port gold coplanar waveguide. The alumina coating acts as a dielectric barrier that suppresses photoluminescence (PL) quenching, while gold nanopillars enhance local electromagnetic fields and induce strain‐driven perturbations of the defect energy levels, causing accelerated photo‐emission. This synergistic effect results in a ∼tenfold enhancement in PL and improves optically detected magnetic resonance to −17% for on‐PNR regions, exceeding comparable prior works by over an order of magnitude. Consequently, we achieve an η_DC_ of 9.4 µT/√Hz, approaching the highest reported values for $V_{B}^{-}$ defects. This research establishes a strategy for designing and fabricating highly sensitive quantum sensors that operate at room temperature without requiring extensive optimization of laser or microwave fields.

## Graphical abstract

Coupling of spin‐active $V_{B}^{-}$ defects in hBN with alumina‐coated gold plasmonic nanoresonators (PNRs), integrated into a microwave‐efficient waveguide, constitutes a plasmon‐strain architecture that enhances their photoluminescence and spin contrast. This platform enables a tenfold quantum yield improvement and achieves a DC magnetic sensitivity (*η*_DC_) of 9.4 µT/√Hz at room temperature without complex field optimization.

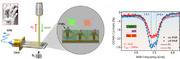

## 1 Introduction

Optically addressable solid‐state spin defects in wide‐bandgap semiconductors, such as nitrogen‐vacancy (*NV* ^−^) centers in diamond [1, 2, 3], silicon vacancies in silicon carbide [4, 5, 6], and donor defects in gallium nitride [7, 8]—have attracted considerable attention for quantum sensing technologies [9, 10, 11]. In principle, these spin defects are expected to exhibit strong spin–photon interfaces without necessitating stringent operational conditions such as cryogenic temperatures, dimensional stripping, or applied magnetic fields. However, as spin defects are deeply embedded in these bulky host materials, they remain physically distant from the target and introduce substantial magnetic noise, reducing the signal‐to‐noise ratio (SNR) [12] and thereby limiting the performance advantages of quantum sensors over their classical counterparts [3].

Hexagonal boron nitride (hBN) is a layered van der Waals two‐dimensional (2D) material, which hosts both the high‐purity single photon emitters (SPEs) [13, 14, 15] and the spin‐active color centers, such as the negatively charged boron vacancy ($V_{B}^{-}$) defects. In particular, the $V_{B}^{-}$ defects embedded in hBN ($V_{B}^{-}$‐hBN) platform benefit from mechanical flexibility and reduced coupling with environmental baths. Nevertheless, the uniqueness of $V_{B}^{-}$ defects lies in its multifaceted sensitivity to optical, thermal, magnetic, and strain fluxes, which enables highly adaptable quantum sensing. In addition, the realization of hybrid 2D heterostructures and the nanometer‐scale proximity of $V_{B}^{-}$ defects to target stimuli uniquely position the $V_{B}^{-}$‐hBN platform for advanced sensing applications [16, 17]. The spin–photon interfaces and spin signatures of $V_{B}^{-}$ defects are optically addressable at room temperature [18] via optically detected magnetic resonance (ODMR) spectroscopy, which employs a combination of laser fluence and microwave (MW) fields [19]. An ensemble of $V_{B}^{-}$ defects can be controllably generated using techniques such as ion implantation, neutron irradiation, and laser writing [20, 21, 22, 23]. In addition, $V_{B}^{-}$‐hBN is compatible with complementary metal‐oxide‐semiconductor (CMOS) platforms [24, 25], enabling precise sensing of temperature [9, 26], strain [9, 27, 28], and magnetic fields [9, 10, 29, 30].

The photoluminescence (PL) associated with $V_{B}^{-}$ defects (∼800 nm) is typically broad due to phonon‐assisted emission, resulting from optically forbidden transitions [31]. This leads to an inherently low quantum yield and diminished sensitivity to external fields [32, 33]. Furthermore, power broadening induced by high laser fluence or MW fields during the ODMR experiments further degrades the emission quality, thereby reducing the SNR [34].

Coupling $V_{B}^{-}$‐hBN with plasmonic structures enables the generation of an intense localized electromagnetic field and subwavelength mode confinement, which collectively enhances both the excitation and spontaneous emission rates through quantum electrodynamic effects [35, 36, 37]. Recent studies have explored this approach by coupling $V_{B}^{-}$‐hBN with nanopatch antennas [38], gold films [29], low‐loss plasmonic cavities [39], and gold nanotrenches [40], all of which demonstrate an enhanced PL. In particular, coupling with protruding nanostructures—such as nanopillars, nanocones, or pyramids—induces strain‐driven lattice symmetry breaking, which modifies the spin Hamiltonian of $V_{B}^{-}$ defects and [27] accelerates their photoemission. For instance, coupling $V_{B}^{-}$‐hBN with SiO_2_ nanopillars has yielded a moderate PL enhancement and an increase in ODMR contrast from −0.5% to −1.4% [28]. Similarly, strain induced by trapped air bubbles at the hBN–gold interface has been shown to enhance PL and shift the ODMR resonance frequency [27]. However, these prior studies have not focused on explicitly addressing improvements in the overall direct current (DC) magnetic field sensitivity (η_DC_) of $V_{B}^{-}$ defects—a critical performance metric for quantum sensing applications; it is expressed as follows [41]:

(1) $$\eta_{DC}=\frac{4}{3\sqrt{3}}\;\frac{h}{g\mu_{B}}\frac{\Delta v}{C\sqrt{I}}$$

Here, *∆ν*, *C*, and *I* denote the ODMR linewidth, ODMR contrast, and PL count rate, respectively; *h* represents Planck's constant; *g* ≈ 2 is the Landé g factor; and *µ*_B_ is the Bohr magneton. Equation (1) indicates that achieving superior η_DC_ requires not only an enhanced PL quantum yield but also improved ODMR contrast (*C*) and reduced linewidth. Although both plasmonic and strain effects can independently enhance PL, their synergistic integration—particularly on plasmonic MW waveguides—remains a significant challenge due to fabrication constraints. Moreover, existing demonstrations of coupling $V_{B}^{-}$‐hBN with nanostructured waveguides typically employ a conventional through‐line (dual‐port) waveguide architecture, which inherently suffers from suboptimal MW absorption, leading to reduced sensitivity in response to subtle resonance frequency shifts [21, 27, 40, 42, 43, 44]. Instead, a single‐port coplanar (gold) waveguide (CPW) design has recently been reported to address this limitation through efficient MW delivery, resulting in an improved ODMR contrast [45]. A unique coupling of strategically fabricated protruding nanostructures within the MW‐efficient single‐port CPW architecture has the potential to substantially enhance the performance of $V_{B}^{-}$‐hBN quantum sensing platforms.

In the present study, we introduce plasmonic nanoresonators (PNRs) to harness plasmon‐strain coupling with $V_{B}^{-}$ defects to enhance the PL and ODMR responses. The PNRs consist of square‐shaped gold nanopillars, coated with a 5‐nm‐thick alumina (Al_2_O_3_), to suppress fluorescence quenching at the interface of $V_{B}^{-}$‐hBN and PNRs [46]. Meanwhile, PNRs provide synergistic in‐plane strain and localized electromagnetic field enhancement, leading to an improved emission from the $V_{B}^{-}$ hBN samples. By leveraging the excellent coupling between $V_{B}^{-}$‐hBN and PNRs, we achieved PL emission exceeding 5 million counts per second and an ODMR contrast of approximately −17%, surpassing previously reported strain‐induced results by an order of magnitude. Consequently, the η_*DC*_ of the hBN quantum sensor was improved to 9.4 µ*T*/√Hz, which is comparable to the highest reported values achieved for $V_{B}^{-}$ defects—obtained using an expensive isotopically purified hBN crystals [47]. Unlike previous strain‐ or plasmon‐only approaches, this work presents an all‐integrated platform that co‐localizes strain, plasmonic nanoantenna enhancement, and on‐chip microwave delivery for advancing chip‐compatible hBN‐based quantum sensing at room temperature.

## 2 Results and Discussion

**FIGURE 1:**
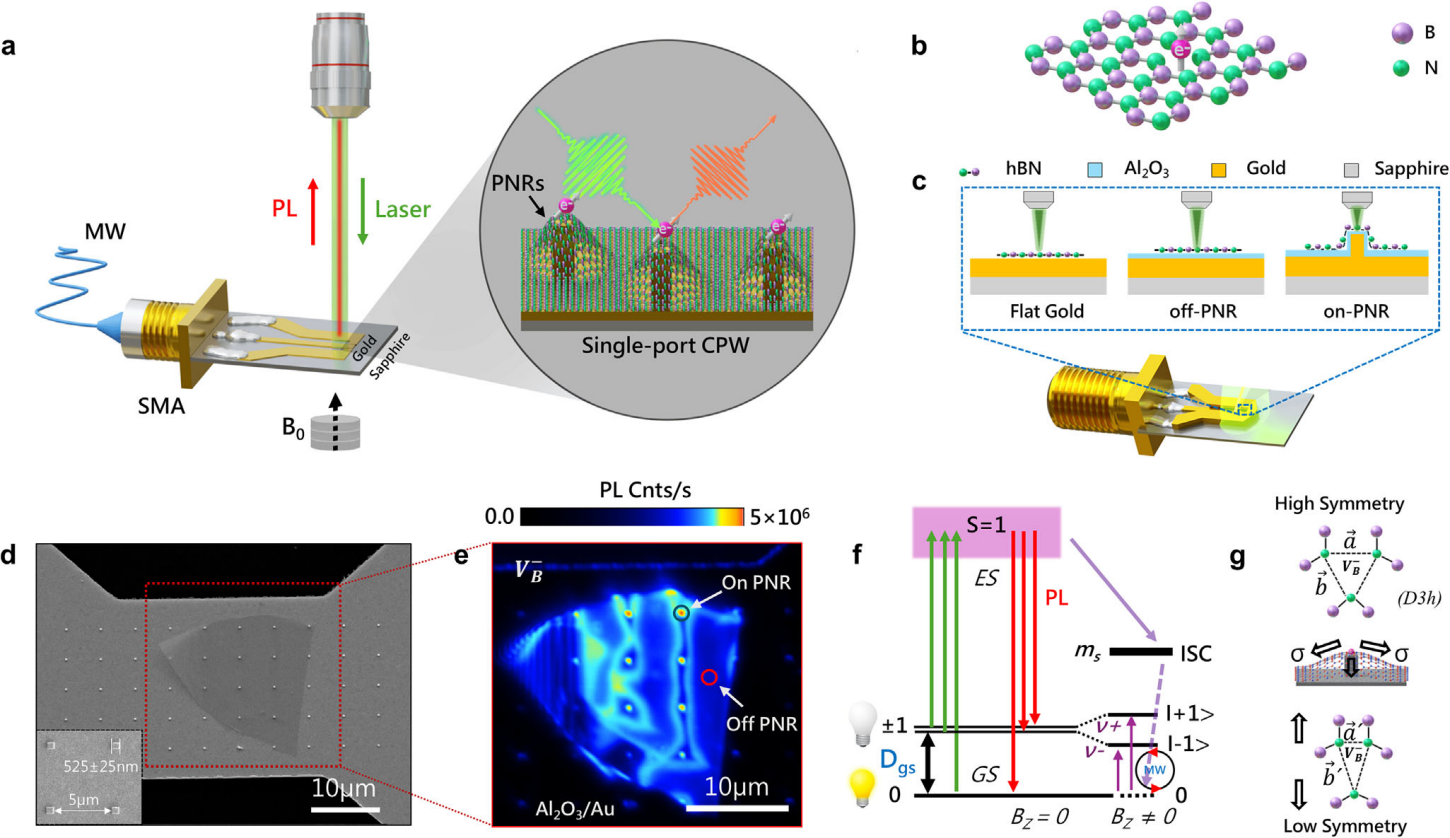
(a) Schematic of the quantum‐sensing prototype device featuring a nanostructured plasmon‐strain CPW coupled to an SMA connector for MW driving. The inset shows a magnified view of the hBN, coupled with PNRs. (b) Atomic structure of a boron vacancy ($V_{B}^{-}$) embedded in the hexagonal lattice of hBN. (c) Illustration of the three waveguide configurations used in this study: flat gold (bare gold without Al_2_O_3_ coating), off‐PNR (flat gold with Al_2_O_3_ coating), and on‐PNR (gold pillars with Al_2_O_3_ coating). (d) FESEM image of an hBN flake deterministically transferred onto the nanostructured (PNR) waveguide. (e) Confocal PL map of $V_{B}^{-}$ defects acquired from the area outlined by the red square in Figure 1d. The red and blue circles denote the off‐ and on‐PNR sites, respectively. (f) Energy level structure of the $V_{B}^{-}$ center, consisting of a triplet ground and excited states. (g) Schematic representation of the hBN lattice with high symmetry (unstrained) with *D3h* point and low symmetry (strained) configurations.

A $V_{B}^{-}$‐hBN quantum sensor device, coupled with a single‐port CPW featuring PNRs, is shown in Figure 1a. The magnified region illustrates $V_{B}^{-}$‐hBN conformally draped over the PNRs, which consist of an array of  Al_2_O_3_‐coated rectangular gold pillars, fabricated on the constricted section of the MW waveguide. The device was fabricated on a sapphire substrate and interfaced with a standard coaxial RF (SMA) connector for MW excitation (see Experimental Section). A 5‐nm‐thick Al_2_O_3_ layer, previously optimized to suppress nonradiative quinching, was deposited via atomic layer deposition on both planar and pillared gold waveguides [48, 49]. A 1–2‐nm‐thick hydrophobic layer of organosilicon molecules was deposited atop the alumina coating to facilitate the transfer of hBN flakes (see Experimental Section). An optical micrograph of the fabricated quantum sensor device integrated with hBN is shown in Figure S1a–c. A schematic representation of a defect embedded within the hexagonal lattice of hBN is shown in Figure 1b. We created an ensemble of $V_{B}^{-}$ defects via helium ion (He^+^) irradiation (see Experimental Section). This study investigated three waveguide design configurations: (1) hBN on bare (flat) gold, (2) hBN on 5‐nm Al_2_O_3_‐coated gold (off‐PNR), and (3) hBN on Al_2_O_3_‐coated PNRs (on‐PNR) (Figure 1c). We used a polymer‐based dry transfer technique to deterministically transfer the $V_{B}^{-}$‐hBN flakes onto the nanostructured CPW for device integration. The detailed transfer procedure is presented in Experimental section. A field‐emission scanning electron microscopy (FESEM) image of a $V_{B}^{-}$‐hBN flake transferred onto the PNRs fabricated on the CPW is shown in Figure 1d. The inset shows the average diameter (D = 520 ± 50 nm) and periodicity (P = 5 µm) of the arrays of fabricated nanopillars on the constricted region of the CPW. Semi‐cross‐sectional and top‐view FESEM images of the nanopillar‐incorporated CPW reveal an average pillar height of 125 ± 25 nm (Figure S2a,b). A confocal PL scan of the $V_{B}^{-}$ defects revealed a pronounced spatial variation in the PL intensity, with enhanced emission observed where the hBN flake conformed to the PNRs (Figure 1e). A peak emission rate exceeding 5 million counts/s was recorded at the on‐PNR site (marked by the blue circle), whereas a significantly lower PL intensity was measured at the off‐PNR (flat) site, indicating strain‐induced enhancement of PL. The energy level diagram of a $V_{B}^{-}$ defect in the absence of external fields, shown in Figure 1f, exhibits a spin‐triplet ground state. This ground state is characterized by a zero‐field splitting of *D/h* = 3.469 GHz between the *m_s_* = 0 and *m_s_* = ±1 spin sublevels, which can be selectively addressed and manipulated using a combination of lasers and MW excitation. Upon laser excitation, the $V_{B}^{-}$ center undergoes one of two primary decay pathways: (1) radiative decay, which produces PL emission in the 800–850‐nm range, and (2) nonradiative decay via intersystem crossing (ISC). The spin Hamiltonian that describes the energy levels of a $V_{B}^{-}$ defect is expressed as follows:

(2) $$H=D\;\left(S_{z}^{2}-\frac{S\left(S+1\right)}{3}\right)+E\left(S_{x}^{2}-S_{y}^{2}\right)+g\mu_{B}\vec{B}.\vec{S}$$

The parameters *D* and *E* represent the zero‐field splitting, *S* denotes the total electron spin (*S* = 1 for triplets), *g* is the Landé factor, *µ*_B_ is the Bohr magneton, *B* is the applied static magnetic field, and *S_x,y,z_* denote the spin‐1 operators.

We considered the strain field as a perturbation, assuming that it is significantly weaker than the dominant Coulombic interactions. In the unstrained state, the lattice exhibits high symmetry with a *D3h* point group (Figure 1g). Owing to the small thickness of the hBN flake, the underlying PNRs primarily induce an in‐plane (transverse) tensile strain (ε_xy_) within the honeycomb lattice. This strain lifts the degeneracy of the m_s_ = ±1 spin sublevels, resulting in an overall shift in the optical transition energies. Additionally, transverse strain causes splitting of the *E_x_* and *E_y_* optical transition.

In our case, strain fields induced by the PNRs introduced displacements Δ*a* and Δ*b* in the lattice parameters, thereby breaking the lattice symmetry of the molecular orbitals and perturbing their energy levels based on H_strain_   =   ∑ i, j σi, j εi, j, where ε*_i,j_* = ∂δ_xi_/∂_xj_ are the components of the strain tensor, and *σ_i_*_,_ *_j_* are the orbital operators [50]. Strain modifies the electronic wavefunctions, thereby enabling optical transitions between the states. This interaction leads to enhanced PL emission [51, 52]. A comparison of the normalized PL spectra of $\;V_{B}^{-}$ defects obtained from both unstrained and strained hBN revealed a considerably broader full width at half maximum (FWHM) in the strained PL peak, indicating the introduction of additional defect states due to strain (Figure S3) [53]. The spectra were acquired using the same laser power. The out‐of‐plane strain (*ε*_zz_) has been reported to primarily induce a pure shift *D*_S_ in ZFS, whereas in‐plane strain affects both the energy level separation and the mixing of new eigenstates *S̃*_x_ and *S̃*_y_ [51, 54]. Regardless of the strain type, the degeneracy of the optical transition is governed by the parameter *E* [21, 51, 55]. The general symmetry‐based spin‐strain Hamiltonian [55, 56, 57] that governs the strain‐induced energy‐level configuration of $V_{B}^{-}$ defects is expressed as follows:

(3) $$\begin{array}{ccc} H_{\varepsilon }=\left[a\left(\varepsilon_{\mathrm{xx}}+\varepsilon_{\mathrm{yy}}\right)+b\varepsilon_{\mathrm{zz}}\right]\;S_{z}^{2} & & +\left[\frac{c}{2}\left(\varepsilon_{\mathrm{xx}}-\varepsilon_{\mathrm{yy}}\right)\right]\left(S_{y}^{2}-S_{x}^{2}\right)\\ & & +c\varepsilon_{\mathrm{xy}}\left(S_{x}S_{y}+S_{y}S_{x}\right) \end{array}$$

Here, S =$\;\Sigma_{i=x,y,z\;}S_{i}$ represents the total spin operator; ɛ denotes the strain tensor; and *a*, *b*, and *c* are the spin‐strain coupling parameters. The coordinates *x* and *y* correspond to the in‐plane coordinates, whereas *z* is aligned with the symmetry axis of $V_{B}^{-}$. In the absence of an external magnetic field, the electron spin resonance can be simplified as a linear combination of zero and non‐zero components of the strain tensor.

(4) $$\hbar \omega =D+D_{s}\pm \sqrt{E^{2}+E_{s}^{2}}$$

(5) $$\begin{array}{ccc} \hbar \omega & & =D+a\left(\varepsilon_{xx}+\varepsilon_{yy}\right)+b\varepsilon_{zz}\\ & & \pm \sqrt{E^{2}+{\left[\frac{c}{2}\left(\varepsilon_{xx}-\varepsilon_{yy}\right)\right]}^{2}+{\left(c\varepsilon_{xy}\right)}^{2}} \end{array}$$

The zero field parameters for our sample were *D/h* = 3.468 GHz and *E/h* = 50 MHz, where *h* is Planck's constant.

**FIGURE 2:**
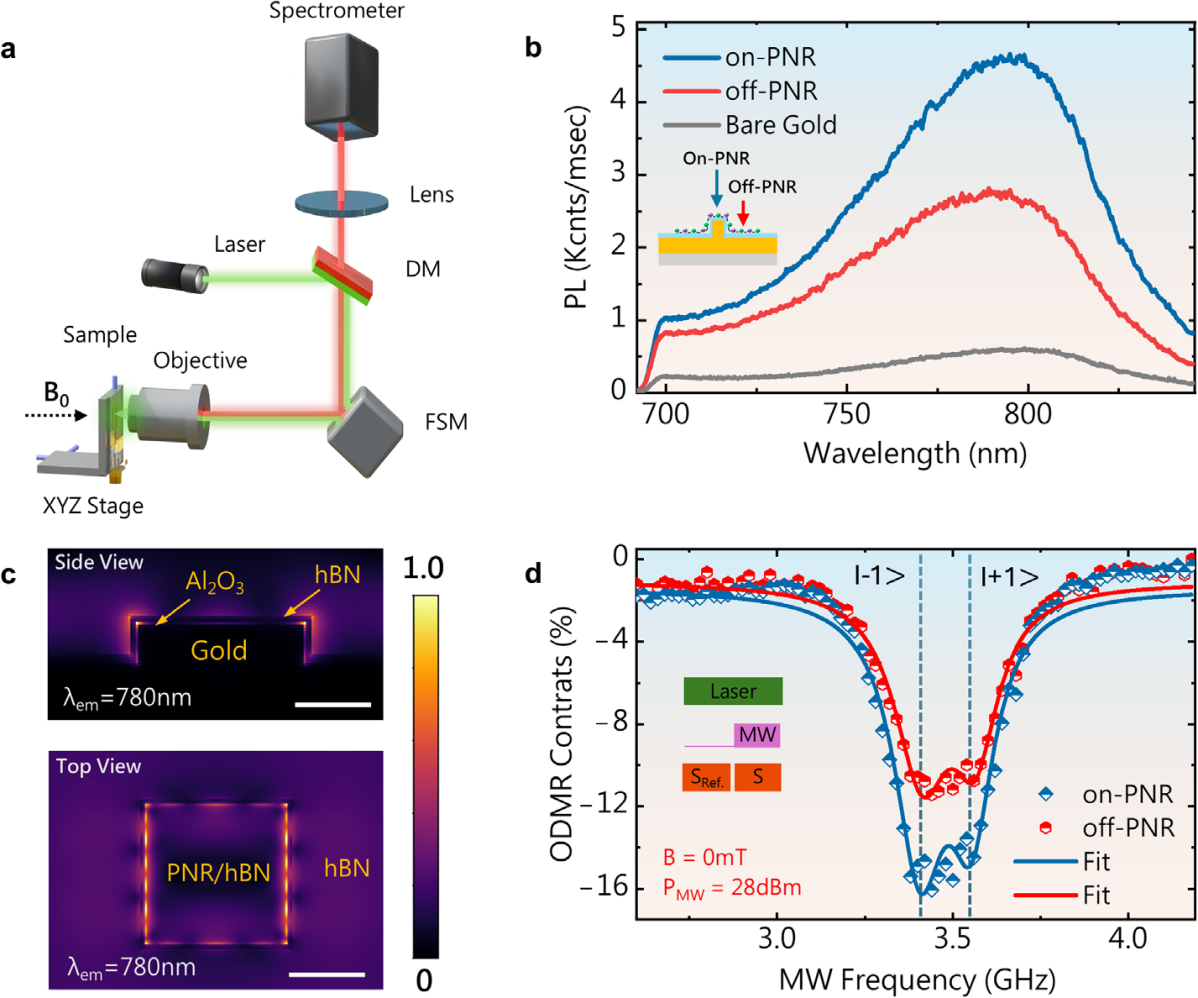
(a) Optical setup used to characterize the hBN quantum sensor. (b) PL spectra of $V_{B}^{-}$‐hBN on bare flat gold (grey), off‐PNR (red), and on‐PNR (blue). (c) FDTD simulation of a PNR, showing the electric‐field localization. The upper image is the side view, while the lower image is the top view of a simulated PNR (d) *cw* ODMR spectra acquired from off (red) and on (blue) PNR, as highlighted in the inset. The measurements were performed at 0 *mT* and at an MW power of 28 dBm.

The optical setup used for continuous‐wave (*cw*) PL and ODMR measurements is shown in Figure 2a. Room‐temperature PL emission spectra of $V_{B}^{-}$ defect ensembles—acquired from flat gold, off‐PNR, and on‐PNR—are shown in Figure 2b. Flake regions located at off‐PNR sites exhibited PL intensities approximately half that of the maximum intensity observed at on‐PNRs. Nevertheless, both on‐ and off‐PNR configurations demonstrated a significant enhancement in PL compared with the PL spectrum obtained from the flat gold sample (without Al_2_O_3_ coating). The PL enhancement observed at the off‐PNR region is primarily attributed to the suppression of radiative losses via a cavity‐like effect induced by the Al_2_O_3_ coating [48]. Supporting this observation, a confocal PL map of $V_{B}^{-}$‐hBN on alumina‐coated flat gold (without nanopillars) is shown in Figure S4a. Flat hBN exhibited modest PL enhancement with the introduction of an Al_2_O_3_ layer compared with flat gold, whereas a sharp increase in PL intensity was also observed in folded hBN regions. By contrast, PL enhancement in the on‐PNR site is driven by a combination of radiative loss suppression and synergistic effects originating from both the strain and localized electric fields introduced by alumina‐coated PNRs. These effects accelerate photoemission [35, 39]. The PL intensities at both on‐ and off‐PNR sites are also plotted as a function of laser excitation power (Figure S4b). The PL from on‐PNR demonstrated a strong dependence on excitation power, with enhanced PL signal intensity, whereas off‐PNR exhibited minimal dependence in the 1–10 mW range, indicating robust sensitivity of the strained regions of hBN to excitation power.

To understand the optical response, we performed finite difference time domain (FDTD) simulations. The results demonstrated that the adopted geometry of the PNR architecture, when coupled with a $V_{B}^{-}$‐hBN flake, supports dual resonances at both the excitation (∼532 nm) and emission (∼800 nm) wavelengths (Figure 2c). These resonances originate from localized surface plasmon resonances of the PNRs (see Text S1) [40]. A key feature of this design is the local enhancement of the pump light, which significantly intensifies the excitation of $V_{B}^{-}$ defects [34], resulting in enhanced fluorescence at those locations. This enhancement is attributed to the plasmonic response of the gold nanopillars, where the resonant mode concentrates the excitation light. The observed double enhancement occurs via two mechanisms: first, by locally amplifying the green pump light, which boosts the excitation rate of $V_{B}^{-}$ defects; second, by facilitating more efficient radiative recombination at the emission wavelength (∼800 nm) owing to a secondary plasmonic resonance mode at this wavelength (See Figure S5a) [58, 59, 60]. The Al_2_O_3_ spacer layer plays a crucial role in mitigating fluorescence quenching through the spatial separation of emitters from the metallic surface, thereby enabling efficient optical coupling of both the pump and emission wavelengths [46, 59]. Our simulations further revealed a spatial map of electric‐field intensity (|E|^2^), showing strong near‐field enhancement localized along the edges of the patterned gold structures beneath the hBN flake (Figure S5b). This near‐field confinement highlights the effective plasmonic coupling achieved by the layered PNR architecture at both the excitation and emission wavelengths.

We performed continuous wave (*cw*) ODMR measurements to identify the ground‐state spin resonances of $V_{B}^{-}$ defects, following the protocol illustrated in Figure 2d. This shows the *cw* ODMR contrast (*C*) measured at zero external magnetic field from both off‐ and on‐PNR regions under an MW power of +28 dBm (see Experimental Section). The ODMR spectra exhibited two resonant dips ν_1_, and ν_2_, corresponding to transitions between the *m_s_* = 0 and *m_s_* = ±1 spin sublevels, manifested as reductions in fluorescence intensity. The central resonance frequency ν_0_ aligns well with the experimentally determined zero‐field splitting (ZFS) value of approximately 3.48 GHz. The ODMR contrast *C* was calculated by normalizing the difference in PL intensity (I) with and without MW excitation as follows: C  =  (I_on _‐ I_off_)/ I_off_. The contrast values achieved for on‐ and off‐PNR were approximately −17% and −11%, respectively, representing a relative enhancement of ∼ 155%. This observed ODMR contrast is nearly an order of magnitude greater than previously reported strain‐driven enhancements [28] and is attributed to strain‐induced symmetry breaking in the *V_B_^−^* defect. This perturbation modifies the relative rates of intersystem crossing between different spin sublevels (*m_s_* = 0 and *m_s_* = ±1) within the spin manifold.

The observed enhancement in ODMR contrast can be attributed to the MW‐efficient single‐port CPW architecture employed in this study. The highest magnetic field intensity at the gold stripline of the device was calculated to be 2520 A/m at a frequency of 3 GHz and an RF power of 1 W. Our simulations showed that most field lines were parallel even up to 30 µm. The impedance profile as a function of frequency showed stable impedance over the working frequency range (2–4 GHz) (device characteristics are shown in Figure S6). Furthermore, we performed simulations to assess the impact of introducing PNRs on the magnetic field intensity at the gold stripline and found no degradation, which confirmed that introducing PNRs does not compromise B field localization (Figure S7). COMSOL Simulations, performed to simulate the MW field distribution along the nanopillars (Figure S8) revealed that the field intensities along the YZ plane are localized at structural boundaries, and significant field magnitudes penetrate the nanopillar regions. Cross‐sectional analysis and field‐line mapping confirm this spatial profile, ensuring efficient magnetic flux delivery to the embedded $V_{B}^{-}$ defect sites.

Understanding the localized PL and ODMR response is essential for effectively coupling strain effects to defect dynamics. We selected four representative points, P1–P4 of PL emission based on the confocal PL intensity profile to examine spatial variations in emission and spin response, as shown in Figure S9a. The PL spectra exhibited the strongest response at P4 (on‐PNR) and the weakest at P1 (off‐PNR). These points correspond to regions experiencing the highest and lowest mechanical strain, respectively. Similarly, *cw* ODMR spectra acquired at these locations (Figure S9b) revealed a gradual increase in contrast from P1 to P4. Furthermore, the MW power was intentionally maintained at the lowest value to allow the observation of local variations in ODMR behavior. The systematic enhancements observed in the localized PL and ODMR suggest a strong dependence on strain in hBN, making it essential to investigate these enhancements using micro‐Raman spectroscopy. Using the PL count rate and ODMR contrast measured on bare gold as a reference (100%), we quantified the additive improvement in $\sqrt{PL}$ (%) as 170% with Al_2_O_3_ coating (off‐PNR) and 158% with nanopillar coupling (on‐PNR). Correspondingly, ODMR contrast showed additive gains of 157% in the off‐PNR region and 155% in the on‐PNR region (Figure S10a). The device demonstrated excellent reproducibility of PL counts measured across multiple nanopillars (Figure S10b,c). In addition, co‐localized PL measurements were used to decouple plasmonic and strain effects, revealing strain as the dominant contributor to PL enhancement compared with plasmonic + Al_2_O_3_ effects (Figure S10d–f).

**FIGURE 3:**
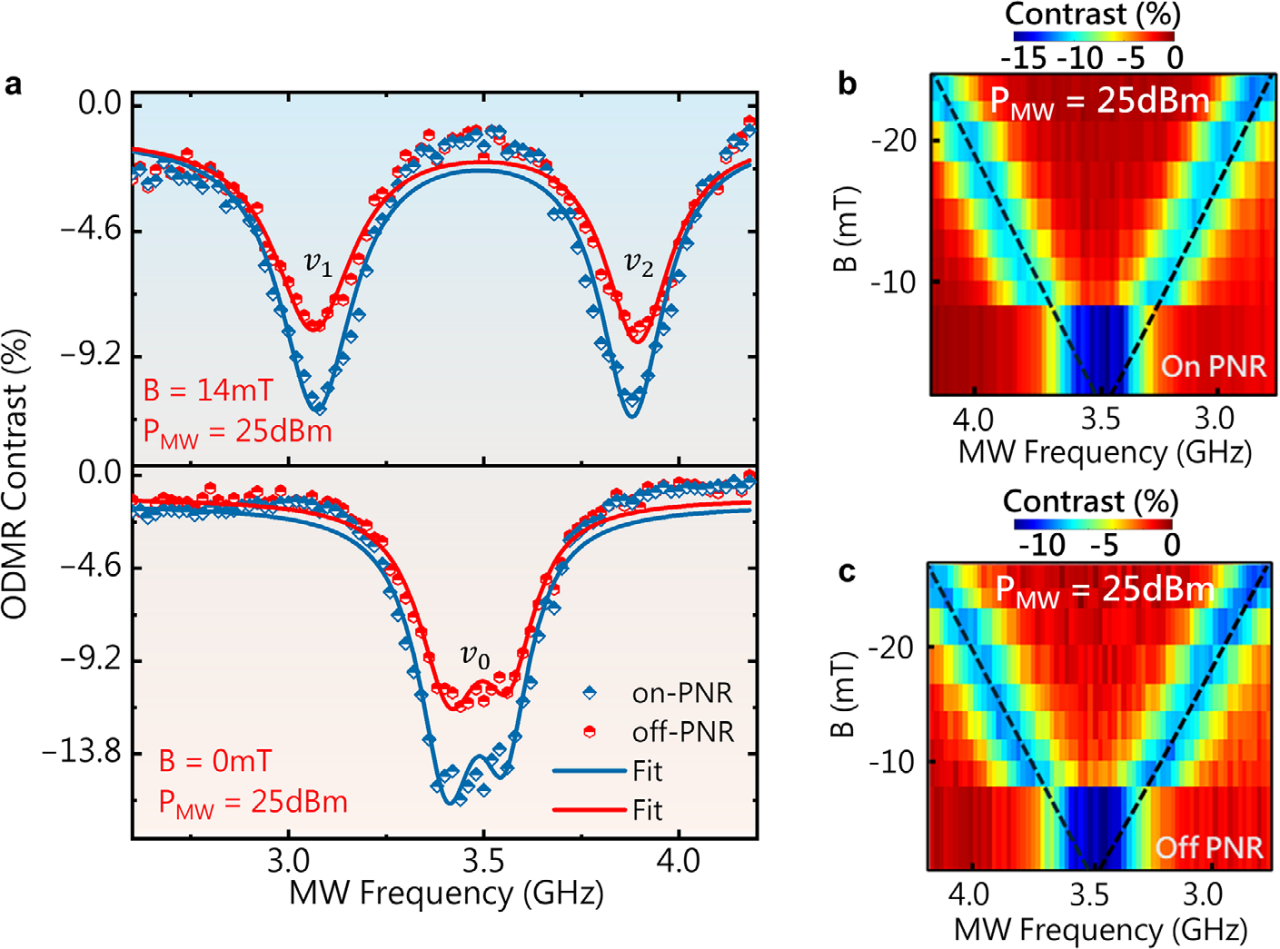
(a) ODMR spectra of both off‐ and on‐PNRs measured at zero magnetic field (bottom) and at an applied magnetic field B = 14 mT (top), showing the corresponding ODMR frequencies ν_0_, ν_1_, and ν_2_. (b) ESR map showing the ODMR frequency as a function of the magnetic field (B || c) for the on‐PNR site. (c) Electron spin resonance (ESR) map shows the ODMR frequency as a function of the magnetic field (B || c) for the off‐PNR site. Experimental data (blue) and the fit (dotted black line) were obtained using Equation (6) using the following parameters: *D/h* = 3.48 GHz, *E/h* = 50 MHz, and *g* = 2.000.

At ambient temperature and in the absence of an externally applied magnetic field, the ODMR spectra of $V_{B}^{-}$ defects at both on‐ and off‐PNRs exhibited two distinct resonance frequencies—ν_1_ and ν_2_—which were symmetrically positioned about a central frequency, ν_0_. This central frequency, ν_0_ corresponds to the axial ZFS parameter D_gs_/*h*, which was quantified as 3.48 GHz. Upon the application of a robust static magnetic field (∼14 m*T*), which was aligned parallel to the hexagonal *c*‐axis, a pronounced splitting between ν_1_ and ν_2_ was observed in the ODMR spectra of $V_{B}^{-}$‐hBN (Figure 3a). The splitting is attributed to the non‐negligible off‐axial ZFS parameter *E_gs_/h*, which was estimated to be 50 MHz. The resonance frequency is expressed as follows:

(6) $$v_{1,2}=v_{0}\;\pm \frac{1}{h}\sqrt{E^{2}+{\left(g\mu_{B}B\right)}^{2}}$$

Here, regardless of the applied magnetic field, strain induction appears to be the primary factor contributing to enhanced sensitivity observed in the on‐PNR region compared with that in the off‐PNR region [9].

A 2D electron spin resonance map, acquired from on‐PNR (Figure 3b) and off‐PNR regions (Figure 3c), showed the ODMR spectral response as a function of MW frequency and an externally applied magnetic field aligned with the *c*‐axis of the crystal. These maps revealed frequency separation between ν*_1_* and ν*_2_*. At all points, C_on‐PNR_ exceeded C_off‐PNR_, and the gap widened at higher MW powers (Figure S11a). The inherently short coherence time of the ensemble typically results in an indistinguishable ODMR spectral line shape [61, 62]. This phenomenon necessitated the application of Lorentzian fitting to resolve individual resonance peaks and, subsequently, to determine the linewidth (∆ν).

(7) $$\left(v\right)=S_{0}-\sum_{m_{I}=\;-3}^{+}\frac{c}{2m_{I}}\frac{{\left(w/2\right)}^{2}}{{\left(v-v_{0}-m_{I}A_{hf}\right)}^{2}+{\left(w/2\right)}^{2}}$$

**FIGURE 4:**
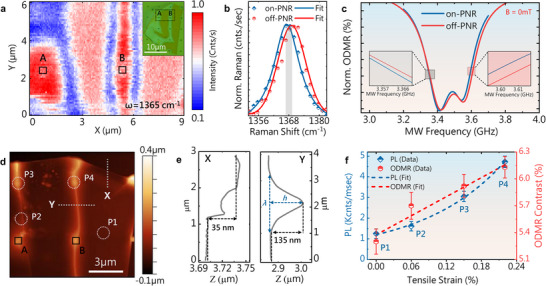
(a) Raman intensity map, acquired at 1365 cm^−1^ from the region highlighted in a dotted rectangle, in the optical microscopy image (inset). (b) Comparative Raman spectra obtained from on‐ (blue) and off‐PNRs (red), showing a tensile strain–driven redshift in the E_2g_ mode in hBN. (c) Normalized ODMR spectra obtained from on‐PNR and off‐PNRs regions showing a strain‐induced shift in the ZFS and resonance frequencies. (d) Atomic force microscopy (AFM) image of the hBN flake on the nanostructured waveguide. (e) Height profiles extracted from line profiles X and Y, showing the flake thickness and wrinkle features. (f) Both the localized PL and ODMR values obtained from points P1–P4 are plotted as functions of tensile strain. The points correspond to the locations highlighted in Figure 4d. Symbols indicate experimental data, whereas the dotted lines represent the fitted curves.

To optimize *η*_DC_, it is essential to maximize the photon count rate and ODMR contrast without substantial power broadening of ∆ν. As shown in Figure S11b, the linewidth as a function of MW power for the on‐PNR configuration, obtained via Lorentzian curve fitting—showed a consistent reduction in power broadening compared with the off‐PNR configuration, although the variation in linewidth largely remained within the experimental error. This suggests that the systematic introduction of PL quenching suppression (by Al_2_O_3_) and strain induction (by PNRs) synergistically improved the photon count rates and contrast, resulting in an overall improvement in the *η*_DC_ of $V_{B}^{-}$ defects, rather than a mere bending‐induced geometric increase in the excitation area, which we estimated to be negligibly small (∼1%) (see Text S2) [28]. By neglecting contributions from technical noise and measurement uncertainty, we used the shot‐noise‐limited sensitivity, as expressed in Equation (1), as a theoretical framework to characterize *η*_DC_. Under similar conditions, the η*_DC_* values for hBN on flat gold, off‐PNR, and on‐PNR sites were estimated to be 91.3, 33.6, and 9.4 $\mu T/\sqrt{Hz}$, respectively (see Figure S12). The optimal *η*_DC_ value of 9.4 $\mu T/\sqrt{Hz}$ was achieved at point P4 (highlighted in Figure 4d in the later section), by optimizing MW power (300 mW), laser intensity (1.5 mW), and measurement conditions under zero magnetic field. This implies that on‐PNR regions achieve nearly an order‐of‐magnitude improvement in magnetic field sensitivity compared with flat gold substrates.

Raman spectroscopy is a powerful, non‐destructive technique for investigating strain by analyzing phonon vibration modes—particularly the in‐plane Raman‐active E_2g_ mode at the Γ point [63, 64]. The Raman spectrometer was initially calibrated using the characteristic Raman peak of crystalline silicon at 520.7 cm^−1^. Comparative Raman analysis revealed that the E_2g_ mode appeared at 1368.1 cm^−^^1^ for the off‐PNR (unstrained) site, whereas a strain‐induced redshift to 1365 cm^−^^1^ was observed at the on‐PNR site. This redshift has been previously attributed to tensile strain in the hBN lattice [27]. Besed on these findings, we performed Raman mapping on a 6 × 9 µm area, encompassing two PNRs (labeled as A and B) under hBN (highlighted in the dotted rectangular block in the inset of Figure 4a. The mapping was conducted by fixing the wavenumber at 1365 cm^−1^. The results provided a spatially distributed strain map, wherein regions exhibiting high strain (red regions) were predominantly localized on PNRs A and B and in the wrinkled channel, in contrast to the surrounding flat regions. Notably, the lattice appeared relatively relaxed directly atop the PNR, likely due to its flat‐top geometry (500 nm wide at the plateau), which facilitates strain relaxation at the center while maintaining high strain accumulation near the edges Figure 4b shows the Raman spectra of off‐ and on‐PNR regions fitted using Lorentzian fitting, demonstrating the redshift cm^−1^. The induced strain was estimated to be approximately 0.23%, which was determined using the following formula:

ε (%)  =  [ω_E2g_ (off‐PNR)  −  ω_E2g_ (on − PNR)] /ω_E2g_ (off‐PNR)  ×  100%

Where ω_E2g_ (off‐PNR) and ω_E2g_ (on‐PNR) are the peak values of the E_2g_ phonon mode corresponding to off‐ and on‐PNR configurations, respectively [65]. However, the actual strain was estimated to be approximately 13.5 cm^−1^/%. Strain fields have been reported to cause shifts in the ZFS and spin resonances [27], which is also evident in the normalized ODMR spectra of both on‐ and off‐PNRs, where a distinct shift to each ODMR resonance for on‐PNR regions and the ZFS can be observed in the insets of Figure 4c. A more systematic shift in *D/h* (ZFS) and *E/h* (spin resonances) is illustrated in Figure S13.

To further quantify strain localization, we performed atomic force microscopy (AFM) to map the surface topography of hBN coupled with PNR arrays (Figure 4d). Line profile X was used to measure the thickness (*t*) of the hBN flake, whereas line profile Y (on the wrinkle) was used to extract the dimensional features, including the height and width of the ridge (Figure 4e). These parameters are essential for estimating the localized strain using the following equation [53, 66]:

(8) $$\varepsilon =\pi^{2}th/\left(1-\sigma^{2}\right)\lambda^{2}$$

Here, *t* represents the flake thickness, *σ* represents Poisson's ratio, and *h* and *λ* denote the height and the width of the ridge, respectively. The values for *h* and *λ* were extracted from line profile B and point z, respectively. The strain, estimated via AFM, was estimated to be 0.238 ± 0.01%, exhibiting close agreement with the Raman spectroscopy–derived strain value of 0.229%.

Tensile strain was also estimated individually at points P1–P4, as shown in Figure 4f. Interestingly, we observed a consistent increase in strain from P1 to P4. This trend is supported by the consistent redshift of the E_2g_ vibration mode from P1 to P4, as shown in Figure S14. Based on these results, a correlation was established by plotting both the PL and ODMR as functions of tensile strain. Both the localized PL and ODMR contrast demonstrated a monotonic increase with tensile strain from P1 to P4.

**FIGURE 5:**
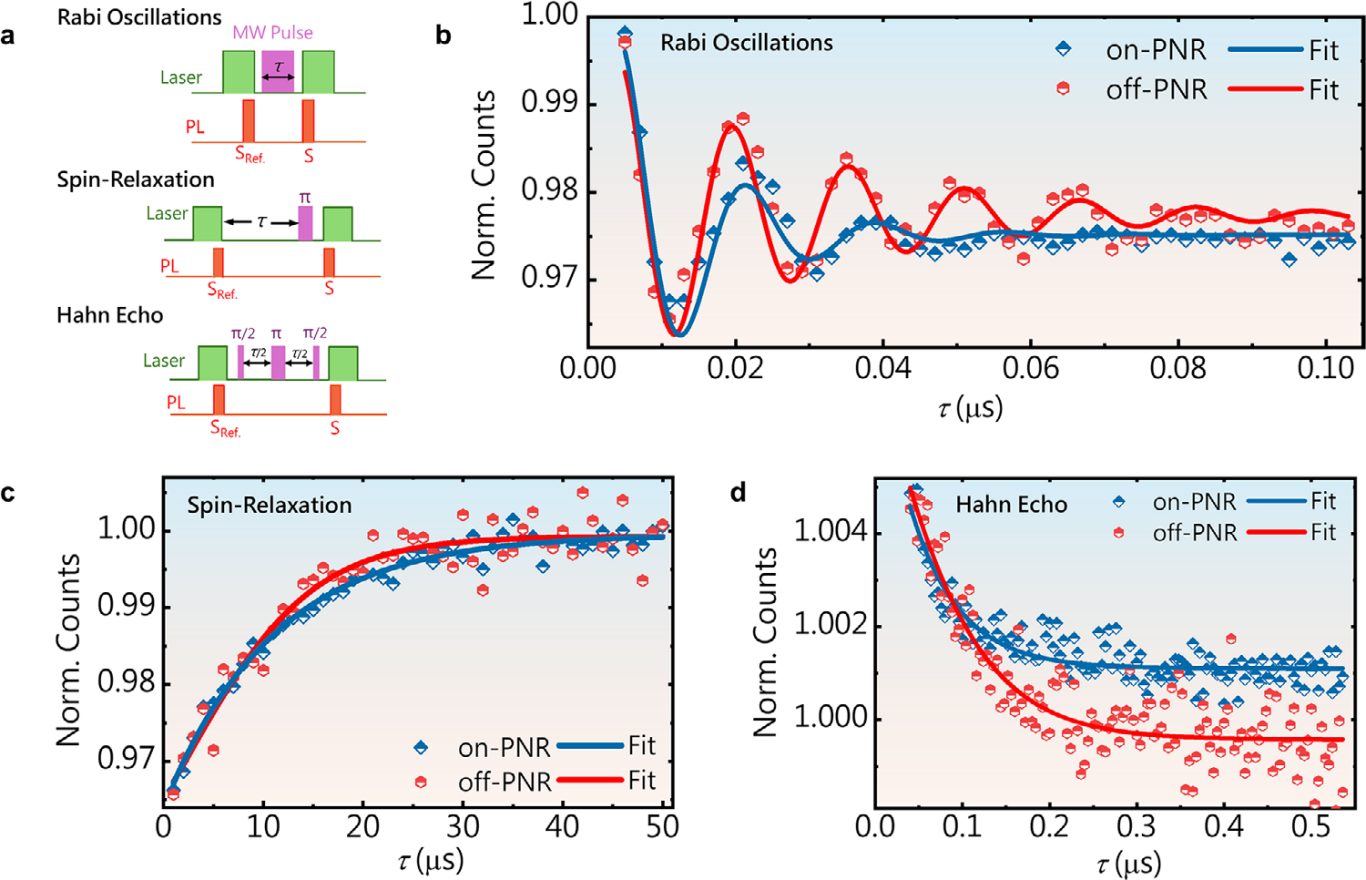
(a) Pulse sequences for Rabi oscillations, T_1_, and Hahn Echo measurement protocols. (b) Rabi oscillation data obtained from off‐PNR and on‐PNR regions. (c) Spin‐lattice relaxation time (T_1_) measurements, performed for off‐PNR and on‐PNR regions. (d) Measurement of coherence time T_2_ using the Hahn echo pulse‐sequence protocol.

The impact of the nanostructured CPW device on spin defect characteristics was assessed using pulsed ODMR measurements. Spin–lattice relaxation (*T*_1_) and spin coherence (*T_2_*) times were quantified across the fabricated hBN‐coupled CPW structures. Figure 5a shows the pulse sequence used for pulsed ODMR measurements, including Rabi oscillation, *T*_1_ relaxation, and Hahn echo measurements. Figure 5b shows the Rabi oscillations recorded for both on‐ and off‐PNR regions. We used a laser pulse to initialize the defects to the *m_s_* = 0 state and to record the PL emission in the *m_s_* = 0 state near the end of the initialization pulse. We then used an MW pulse with a fixed power and varying duration τ to drive the transition between *m_s_* = 0 and *m_s_* = −1 states. The resulting oscillations in PL contrast, defined as *PL*_*MW* *ON*_/*PL*_*MW* *OFF*_, are plotted. The data were fitted to the function Asin(ωt+ϕ)exp(−t/τ)+c to extract the Rabi oscillation frequency and spin dephasing time *T*_2_ ^*^. We observed two important effects: (1) the contrast at longer pulse durations *τ* was lower at the on‐PNR region than the off‐PNR region, and (2) Rabi oscillations at the on‐PNR region were slower than those in the off‐PNR configuration. This suggests that the MW power delivery is slightly less efficient at the on‐PNRs, attributed to the quantization axis of the defects, which lies along the *c*‐axis of the crystal and is perpendicular to its surface. In other words, *the reduced pulsed contrast and T_2_ on‐PNRs arise from the curved hBN geometry, which introduces an angular distribution of V*_B_^−^ *defect axes, leading to nonuniform microwave driving and increased susceptibility to magnetic noise*. This is in contrast to the section of the flake lying flat on the substrate, which receives a better‐aligned MW magnetic field. This explains the reduced contrast and the smaller *T*_2_ as observed on‐PNR in Figure 5b. The Rabi frequencies under identical conditions were 56 and 63 MHz at on‐ and off‐PNRs, respectively, which further supports the above argument.

Figure 5c shows the spin decoherence time *T*_1_ measurements for both on‐ and off‐PNR regions. We initialized the spins into the *m_s_* = 0 state and recorded the corresponding PL emissions. The system was allowed to relax for a variable time interval *τ*, during which spin relaxation occurred. A *π* pulse—whose duration was determined from the Rabi oscillations—was then applied to flip the spin state to m_s_ = −1 for the *T*_1_ measurement. The resulting data followed an exponential decay curve, as shown in the figure. The measured *T*_1_ values were 11.18 and 9.39 µs for the on‐PNR and off‐PNR regions, respectively. This modest increase in *T*_1_ can be attributed to local variations in *T*_1_ within the flake, which may arise from minor differences during crystal growth or the doping process. Figure 5d shows the spin coherence time *T*_2_, measured via the spin echo pulse sequence. The spin was initialized into a coherent superposition of *m_s_* = 0 and *m_s_* = 1 states via a π/2 pulse, the duration of which was derived from the Rabi oscillation. After allowing the spin state to evolve freely for a duration τ, a *π* pulse was applied to flip the spin trajectory, followed by another π/2 pulse at time 2τ. The final readout was performed using a laser pulse. This resulting signal exhibited an exponentially decaying curve, the decay constant of which represents the spin coherence time *T*_2_. The measured *T*_2_ values were 57.68 and 74.65 ns for the on‐PNR and off‐PNR regions, respectively. This reduction in *T*_2_ for the on‐PNR region can be attributed to the optimization of MW pulses for on‐PNR measurements. Rabi oscillations were calibrated on flat regions to define the *π*‐pulse, and the reduced on‐PNR Rabi frequency confirms the geometric misalignment of defect axes with the microwave field due to conformal hBN curvature. Consequently, the π pulse duration derived from these oscillations will be an aggregate of the ensemble and will not be the same for all defects present under the laser spot. This indicates that the pulses applied during *T*_2_ measurement will further reduce the coherence time by introducing additional noise for non‐optimized defects in the ensemble, ultimately reducing the coherence time. The slight reduction in the Rabi coherence and the spin coherence times is offset by the fact that the quantum yield of the emitters is enhanced several times. A variation in the sensitivity with 1/√I improves local magnetic field measurements with spin defects in hBN. This leads to faster acquisition times and brings us closer to the real‐time monitoring of environmental magnetic fields surrounding the flake. Polarization‐resolved PL across multiple regions on $V_{B}^{-}$‐hBN reveals a marginal dependence on the polarizer axis, which is consistent with the inherent instrumental dichroic effects rather than intrinsic defect symmetry (Figure S15) (see Text S3).

**FIGURE 6:**
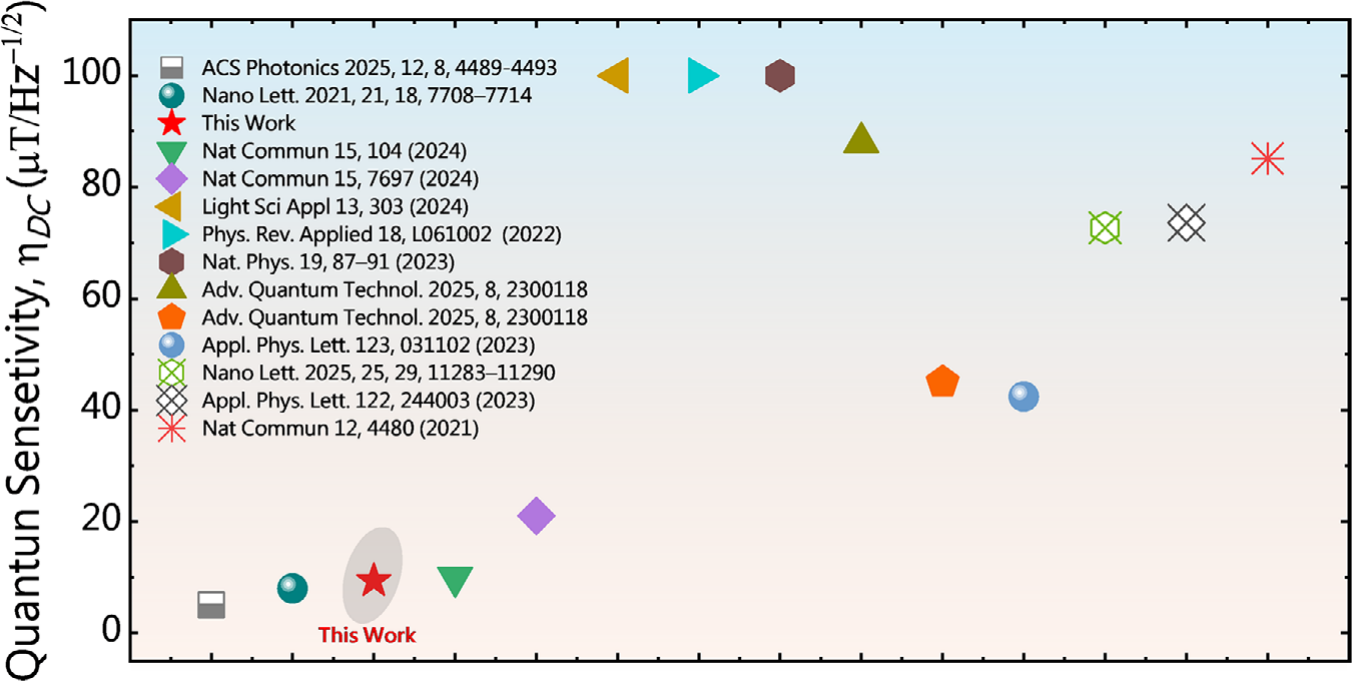
An overview of the η_DC_ values reported to date for the $V_{B}^{-}$ ensemble in hBN.

A comparative overview of the η_DC_ values reported for the $V_{B}^{-}$ ensemble in hBN to date, including data from isotopically purified hBN crystals as well as recent studies published during the preparation of this manuscript, has been presented in Figure 6. Notably, our results demonstrate a high η_DC_ achieved solely through the design of nanostructured waveguides, without the need for focused optimization of laser or microwave fields. Given the strong evidence of coupling between PNRs and $V_{B}^{-}$ defects observed here, further systematic investigations are warranted to deepen the understanding and control of the spin properties of $V_{B}^{-}$− defects in hBN.

## 3 Conclusions

In conclusion, we introduced a nanostructured plasmonic single‐port microwave waveguide design that leverages arrays of PNRs to significantly enhance the quantum yield (PL) of the $V_{B}^{-}$ ensemble in hBN. We investigated three waveguide configurations: hBN coupled with flat bare gold, off‐PNR, and on‐PNR. Compared with flat gold, the introduction of an Al_2_O_3_ spacer on the waveguide mitigated fluorescence quenching, yielding a noticeable improvement in PL. However, the most substantial enhancement was achieved through the synergistic interplay of PNR‐induced in‐plane lattice strain and localized plasmonic enhancement when the $V_{B}^{-}-$ hBN was coupled with PNR arrays. This enhancement was further validated by ODMR spectroscopy, which revealed a clear dependence on strain. To quantify this strain, micro‐Raman spectroscopy and AFM analyses were performed, which revealed a strong correlation between the induced strain, observed PL intensity, and ODMR contrast specifically, for both on‐ and off‐PNR regions. The PL intensity and the ODMR contrast demonstrated a monotonic increase with increasing induced in‐plane strain, resulting in an overall enhancement in η_DC_. These findings demonstrate that PNR waveguides offer a robust and CMOS‐compatible platform for manipulating and enhancing the optical properties of defects, contributing to the development of highly sensitive quantum sensors. The strategic design of PNRs, including their geometry and periodicity, enables the investigation of material properties at highly localized scales with an enhanced SNR, rendering this technique vital for quantum sensing applications.

## 4 Experimental Section

### 4.1 Nanostructured Waveguide Device Fabrication

The fabrication protocol for the quantum sensing device was informed by computational modeling results. A *c‐*plane (0001) sapphire substrate with 0.2 ± 0.1° off the *M*‐axis was used for depositing a gold (Au) waveguide. The process began with a cleaning step, where the samples were placed in heated acetone (45°C) for 15 min with ultrasonication, then rinsed in isopropyl alcohol (IPA) and dried. Before spin‐coating the e‐beam resist, an oxygen plasma treatment was performed for 2 min. The samples were then coated with a layer of PMMA 950K A6, 700 nm thick, and baked at 180°C for 3 min. A thin layer of the conductive polymer Espacer was spin‐coated on top of the resist to avoid charging effects. The pattern was aligned with the global marks of the first layer and exposed using a 100 kV e‐beam lithography system with a beam current of 2 nA and an area dose of 1200 µC/cm^2^. The exposed PMMA was developed in a 1:3 solution of methyl isobutyl ketone (MIBK) and IPA for 2 min, followed  by rinsing the samples in IPA for 30 s. Before metal deposition, an oxygen plasma treatment at low power was performed for 20 s to remove residual PMMA from the open holes. Then, 10 nm of Ti and 300 nm of Au were deposited on the samples using e‐beam evaporation. The final pattern transfer was completed through a lift‐off process, in which the sacrificial PMMA layer was dissolved in acetone, assisted by heating and ultrasonication. The Ti/Au bilayer constituted a single‐port coplanar waveguide (CPW) architecture, designed for the controlled delivery of microwave signals within the device.

### 4.2 Atomic Layer Deposition of Alumina on the Waveguide

A roughly 5 nm‐thick Al_2_O_3_ layer was deposited on the substrate using atomic layer deposition (ALD) at 180°C and 920 mTorr, employing trimethylaluminum (TMA) and water as precursors. Subsequently, a hydrophobic treatment was applied via molecular layer deposition (MLD) at 50°C and ∼744 mTorr, utilizing organosilicon molecules and water as precursors. Forty cycles were performed for both the ALD and MLD processes, resulting in average film thicknesses of 4.8±0.2 nm and 1.5±0.2 nm for the Al_2_O_3_ layer and the hydrophobic coating, respectively, as measured by ellipsometry.

### 4.3 $V_{B}^{-}$ defect Implantation in hBN

The creation of spin‐active $V_{B}^{-}$ ensemble in h‐BN was performed using the ion‐implantation method. The hBN flakes were mechanically exfoliated on to a 10 mm × 10 mm Si/SiO_2_ substrate. Controlled helium ion (He^+^) irradiation (with an optimized ion‐beam energy), with a fluence of approximately ∼10^13^–10^14^ ions/cm^2^ and exposure times of 300–400 s, was employed to deterministically create $V_{B}^{-}$ ensembles. No post‐fabrication annealing was performed before transferring hBN flakes onto the nanostructured waveguide.

### 4.4 Deterministic Transfer of hBN Flake on Nanostructured Waveguide

The $V_{B}^{-}$‐implanted hBN flakes were transferred from Si/SiO_2_ onto the nanostructured waveguide using a polymer‐assisted transfer technique [29]. A polycarbonate (PC) film, supported by a polydimethylsiloxane (PDMS) substrate, was employed as a transfer stamp for hBN flake transfer. The stamp was positioned via a micromanipulator‐integrated optical microscope system 2D Heterostructure Transfer System, hq graphene) affixed to a glass substrate. The Si/SiO_2_ substrate, containing $V_{B}^{-}$‐implanted hBN flakes, was secured firmly on a heated stage via vacuum suction. The PC stamp was brought into contact with selected flakes. The stage temperature was regulated at 145°C for 10 min, followed by passive thermal relaxation. The PC stamp was lifted when the temperature reached 60°C, carrying flakes with it. The Si substrate was removed, and the target substrate (nanostructured waveguide on sapphire) was placed on the heating stage. The flakes were positioned over the constricted part of the waveguide. The PC stamp and target substrate were brought into conformal contact. The stage was heated to 160°C and then cooled gradually. The glass slide with the PDMS block was then detached, leaving the PC stamp adhered to the nanostructured waveguide. The PC stamp remained on the target substrate. Chloroform, acetone, and isopropyl alcohol were used sequentially to dissolve the PC stamp and clean the substrate surface.

### 4.5 Confocal Microscopy Measurements

Optical characterization was performed at ambient temperature using a custom‐built confocal microscopy system. A 532 nm laser source was directed via a 650 nm dichroic mirror and subsequently focused on the sample utilizing a 100x objective lens with a high numerical aperture (NA = 0.9). Acousto‐optic modulation (ISOMET, M1205‐T110L‐1) was used for rapid optical switching. Photoluminescence (PL) was isolated from the excitation laser using a dichroic mirror, with residual laser light suppressed by two 550 nm long‐pass filters. The PL signal was then coupled into a 125 µm single‐mode fiber and routed to either a single‐photon detector (Excelitas, SPCM‐AQRH) or a spectrometer (Andor Shamrock SR‐500).

### 4.6 ODMR Measurements

Continuous wave (cw) optically detected magnetic resonance (ODMR) measurements were performed using a custom confocal microscope, employing a 532 nm laser, a 550 nm dichroic mirror, a 100x, NA 0.9 objective, and an acousto‐optic modulator (ISOMET, M1205‐T110L‐1) for optical switching. The PL was separated from the laser by the dichroic mirror, and any residual laser light was blocked by two 550‐nm long‐pass filters. The PL was coupled into an optical fiber and directed to a single‐photon counter (Excelitas, SPCM‐AQRH). Microwaves were generated by a Stanford Research Systems SG386 signal generator. The amplitude was modulated by two fast RF switches (Mini‐Circuits ZASWA‐2‐50DRA+) and then amplified by an amplifier (Mini‐Circuits ZHL‐10W‐202s or ZHL‐16W‐43‐s+). For ODMR measurements, a pulse streamer (Swabian Instruments Pulse Streamer 8/2) sent pulses to modulate the RF switches, signal generator, and AOM. A permanent magnet mounted on a translation stage, combined with a goniometer behind the sample, enabled the application of a tunable external DC magnetic field.

## Funding

This work was solely funded by Toyota Motors Corporation, Japan.

## Conflicts of Interest

We would like to declare an in‐process submission of an intellectual property (IP) patent, to be filed in the coming days. We're happy to provide more details if required.

## Supporting information




**Supporting File**: adma72797‐sup‐0001‐SuppMat.docx.

## Data Availability

The data that support the findings of this study are available from the corresponding author upon reasonable request.
