# Supplementary material for: Coupling Nanostructured Plasmon–Strain Microwave Waveguide to Spin Defects in Hexagonal Boron Nitride for High‐Sensitivity Quantum Sensors
Source: Adv Mater. 2026 Mar 28;38(24):e16761. doi: 10.1002/adma.202516761 (PMC13113239; doi:10.1002/adma.202516761)
Supplement: Supplementary file 1 — Supporting File: adma72797‐sup‐0001‐SuppMat.docx. [file ADMA-38-e16761-s001.docx]

Supporting Information

Coupling Nanostructured Plasmon–Strain Microwave Waveguide to Spin Defects in Hexagonal Boron Nitride for High-sensitivity Quantum Sensors

*Naveed Hussain,^1^* Sumukh Vaidya,^2^ Saakshi Dikshit,^3^ Shahriar Esmaeili,^1^ Paul Schmalenberg,^1^ Hayate Yamano,^4^ Katsunori Danno,^4^ Biswajit Sahoo,^5^ Shougo Higashi,^1^ Ercan M. Dede,^1^ Tongcang Li,^2,3^ Debasish Banerjee,^1^* *Songtao Wu,^1^**

***Contents***

**Text S1**. FDTD simulations of $V_{B}^{-}$-hBN coupling with PNRs

**Fig. S1:** The hBN Quantum sensor, showing hBN coupled with PNRs

**Fig. S2:** FESEM images showing semi-cross-sectional and top views of gold nanopillars fabricated on a waveguide.

**Fig. S3:** Normalized PL spectra, obtained from on- and off-PNR, showing FWHM of their PL peaks.

**Fig. S4:** **(a)** Confocal PL map of $V_{B}^{-}$ defects over a flat Al_2_O_3_-coated waveguide, without nanostructures. **(b)** PL intensities of $V_{B}^{-}$ defects on- (blue) and off-PNR (red) as functions of the laser power.

**Fig. S5:** **(a)** The normalized extinction spectrum reveals the wavelength-dependent plasmonic resonance characteristics of PNRs. The inset shows an enlarged image of the e-field concentration at the PNR. **(b)** The edge-driven strain enhancement in $V_{B}^{-}$ PL.

**Fig. S6:** **(a)** COMSOL simulations of a planar coplanar waveguide (CPW) showing field accumulation. (**b**) The quantification of the field accumulation at the constricted part of the CPW using COMSOL simulations. (**c**) The impedance characteristics of CPW as a function of frequency. **(d)** B-field accumulation at the constricted part of the CPW at various MW frequencies (2-4 GHz), showing the optimal results around 3GHz.

**Fig. S7:** **(a)** COMSOL simulations of a planar coplanar waveguide (CPW) showing field accumulation. (**b**) COMSOL simulations were performed for the nanostructured waveguide using the same parameters, showing almost no effect on field accumulation after introducing PNRs onto the CPW.

**Figure S8:** (**a**) shows the Magnetic field distribution in the y-z plane of the waveguide. **(b)** shows the zoomed-in figure showing the magnetic field concentration in the region around the nanopillars. Inset c) shows the field line distribution of the magnetic field. While the maximum magnetic field is concentrated on the edges, the region near the nano pillars also experiences a significant field.

**Fig. S9:** **(a)** Localized PL spectra acquired from points P1-P4, highlighted by yellow circles in the inset (f) *cw* ODMR spectra obtained from points P1-P4, showing strong correlation with the amount of strain. The ODMR spectra were acquired at a MW power of 15 dBm and in the presence of an external magnetic field of 7 mT.

**Fig. S10:**  (**a)** Additive gains (%) in √PL and ODMR contrast from pure plasmon, plasmon + Al₂O₃ coating (off-PNR), and subsequent nanopillar integration (on-PNR). **(b)** PL map of the hBN flake. The associated measurement uncertainty is ±1.**(c)** Histogram of the associated measurement uncertainty is ±1. **(d)** AFM topography image with corresponding height profile. **(e)** Co-localized PL of VB⁻ defects from the flake in (d). **(f)** PL spectra acquired at points 1–4 highlighted in (d,e).

**Fig. S11**. (**a**) MW power dependence of ODMR contrasts for on- and off-PNRs, measured at a magnetic field of 30 mT. (**b**) Variation in the ODMR contrast linewidth (∆υ) from on- and off-PNRs with varying MW power levels to demonstrate power broadening.

**Fig. S12**: The DC magnetic field sensitivity (*η*_DC_) achieved for $V_{B}^{-}$-hBN lying over bare gold, off-PNR, and on-PNR.

**Figure S13:** Strain-driven ZFS and spin resonance shift in normalized ODMR spectra, obtained from on- and off-PNR. The inset shows full spectra.

**Figure S14:** (**a**) The optical micrograph of the hBN-coupled PNRs, showing points P1-P4. (**b**) Corresponding redshift in E_2g_ mode of hBN, showing an increasing tensile strain from P1-P4. Strain-driven ZFS and spin resonance shift in normalized ODMR spectra, obtained from on- and off-PNR. The inset shows full spectra.

**Fig. S15:** Polarization dependence of the emission from 6 different spots on the sample.

***Text S1: FDTD simulation of hBN-PNRs***

Finite-difference time-domain (FDTD) simulations were conducted using the tidy3d ^[1]^ FDTD package to investigate the scattering characteristics and near-field electromagnetic response of the hBN-draped plasmonic nanopillar (PNR) system. A plane wave excitation was implemented to illuminate the structure with a transverse-magnetic (TM) polarized plane wave, incident normal to the hBN-PNR interface. The simulation domain was three-dimensional, sized to 5 μm in each direction, discretized using a cuboid mesh, with a general size of 20 nm in the x-, y-, and z-directions. Mesh density near the pillar increased to 2.5 nm to ensure high spatial resolution in regions of strong field confinement near material interfaces for a total of 29 million mesh cells.

The frequency-dependent optical constants for alumina, ^[2]^  gold, ^[3]^ and hBN were incorporated via experimentally tabulated refractive index data. Perfectly matched layer (PML) boundary conditions were applied in all spatial directions to eliminate spurious reflections and emulate an open system. Frequency-domain field and power monitors were placed along the material interfaces and selected cross-sectional planes to quantify near-field electric field enhancements at both resonant and off-resonant wavelengths. Flux monitors were used to quantify the local enhancement near the pillar for comparison to a reference simulation without the pillar.

In particular, |E|^2^ enhancement plots (e.g., at 536 nm) were extracted to visualize strong plasmonic hotspots concentrated near the edges of the gold structures, where the electromagnetic field is intensified due to resonant confinement. The excitation spectrum spanned a wavelength range from 500 nm to 900 nm, encompassing both the pump (green) and emission (near-infrared) wavelengths relevant to boron vacancy defect photoluminescence in hBN.

(1). Flexcompute. Python-Driven FDTD Software: Tidy3D Flexcompute. Flexcompute.com. https://www.flexcompute.com/tidy3d/solver/ (accessed 2025-05-14).

(2). Lorentz Dispersion Model. https://www.horiba.com/fileadmin/uploads/Scientific/Downloads/OpticalSchool_CN/TN/ellipsometer/Lorentz_Dispersion_Model.pdf (accessed 2025-05-14).

(3). Olmon, R. L.; Slovick, B.; Johnson, T. W.; Shelton, D.; Oh, S.-H.; Boreman, G. D.; Raschke, M. B. Optical Dielectric Function of Gold. Physical Review B 2012, 86 (23). <https://doi.org/10.1103/physrevb.86.235147>.

***Text S2: Effect of additional area due to tentpole geometry under laser excitation.***

We can do a simple estimation of the increase in area under laser excitation both on and off the pillar, as seen in the figure below. The laser spot is approx. 1$\mu$m in diameter, and from the AFM topography data, the angle of the hBN with respect to the flat surface is approximately:

$${\theta= tan}^{-1} \frac{135 nm}{1000 nm}={7.6}^{o}$$

From this, we can calculate the increase in area $\sec\theta=1.009$ , since the fold is along a single direction instead of a bubble. The scale bars on the Z and the X, Y axes are different to highlight the folding of the hBN nanosheet lying on top of nanopillars.

This provides an increase in area of approximately 1%. Under the assumption that PL scales linearly with surface area, this would result in a marginal 1% enhancement in the PL intensity. This contribution is negligible compared to the more than 150% increase observed between the on- and off-PNR cases, suggesting that the enhancement is primarily driven by strain-induced effects, and localized field enhancement, rather than simple geometric scaling.

Since the ODMR contrast is defined as the ratio $\frac{n_{photon}\left( MW ON \right)}{n_{photon}(MW OFF)}$, the total contrast seen in an ensemble is not dependent on the number of emitters contained in the region of interest. The number of emitters only influences the total number of photons received, which will have implications for readout.


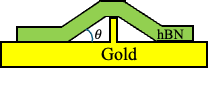


***Text S3: Emission polarization studies of*** $\boldsymbol{V}_{\boldsymbol{B}}^{\boldsymbol{-}}$

Due to the ensemble nature of the $V_{B}^{-}$ spin defects, with typically ${10}^{5}$ defects under the laser spot, emission polarization dependence is not expected from these defects. We have performed emission polarization studies and are including the data from 6 points on the sample here in Figure S.14. We observe slight dependence on the polarizer axis with respect to the confocal system horizontal axis, across all points. This can be explained in terms of our optical system, where we have several filters and mirrors in the optical path. Due to polarization dependence of dichroic mirrors and mirrors where the transmission and reflection differ by a few percent for s and p polarizations, we see a slight dependence on the axis across all the spots. This does not affect the spin properties of the $V_{B}^{-}$ spin defects as we observe from the high ODMR contrast.


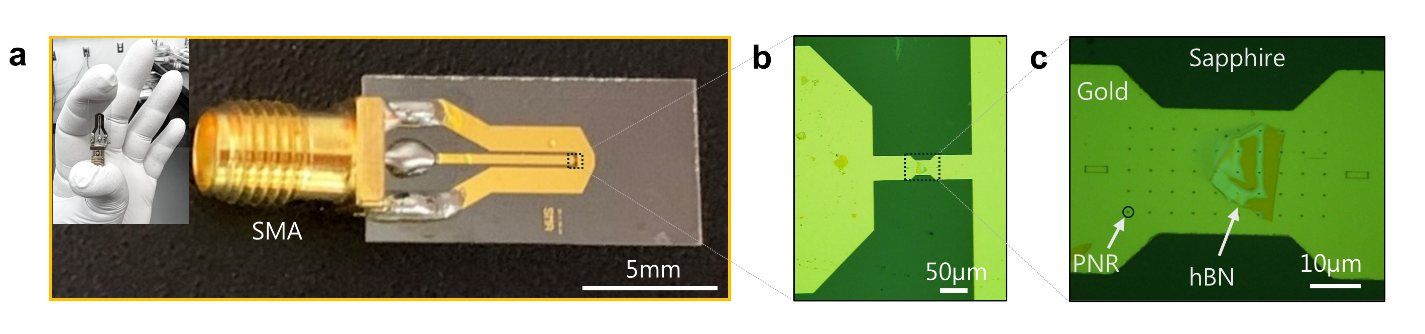


**Fig. S1**. (**a**) A compact hBN quantum sensor with a nanostructured waveguide. The inset shows the compact size of the hBN quantum sensor. (**b**) A zoomed-in optical micrograph of the hBN integrated plasmonic waveguide. (**c**) Highly zoomed-in micrograph showing the $V_{B}^{-}$-hBN coupled with an array of plasmonic nanoresonators (PNRs).


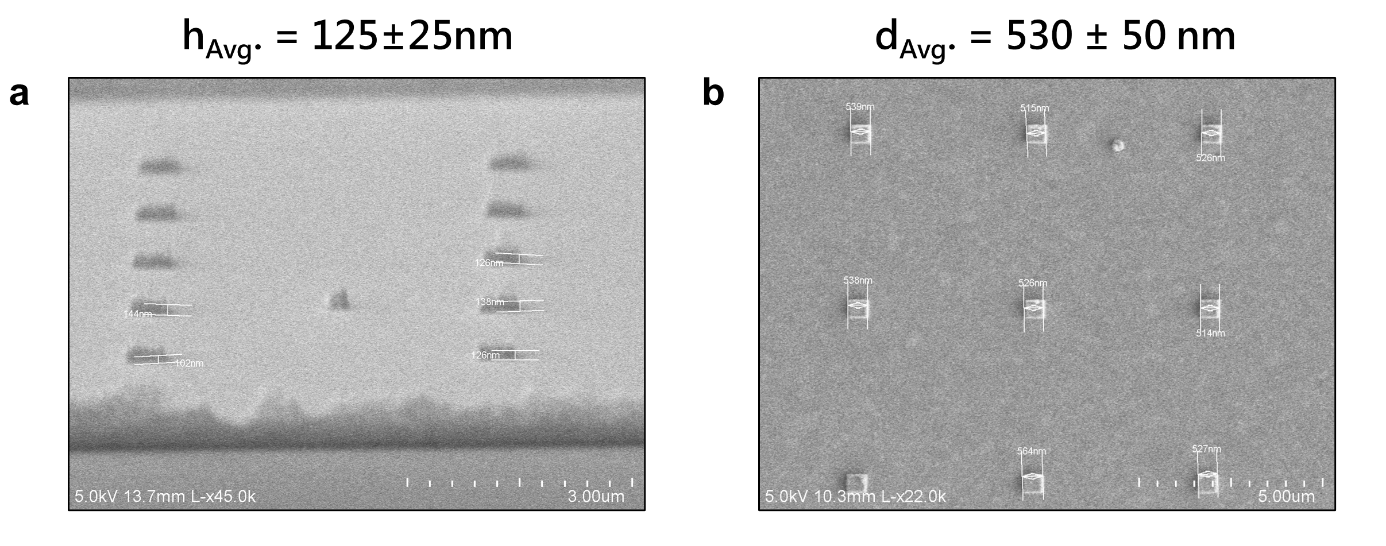


**Fig. S2**. **(a)** FESEM image of the nanostructured waveguide, captured at a tilted view of 45^0^, showing the average pillar height, mentioned on top. **(b)** The FESEM image of the same waveguide from the top, showing square-shaped features and spacing of PNRs.


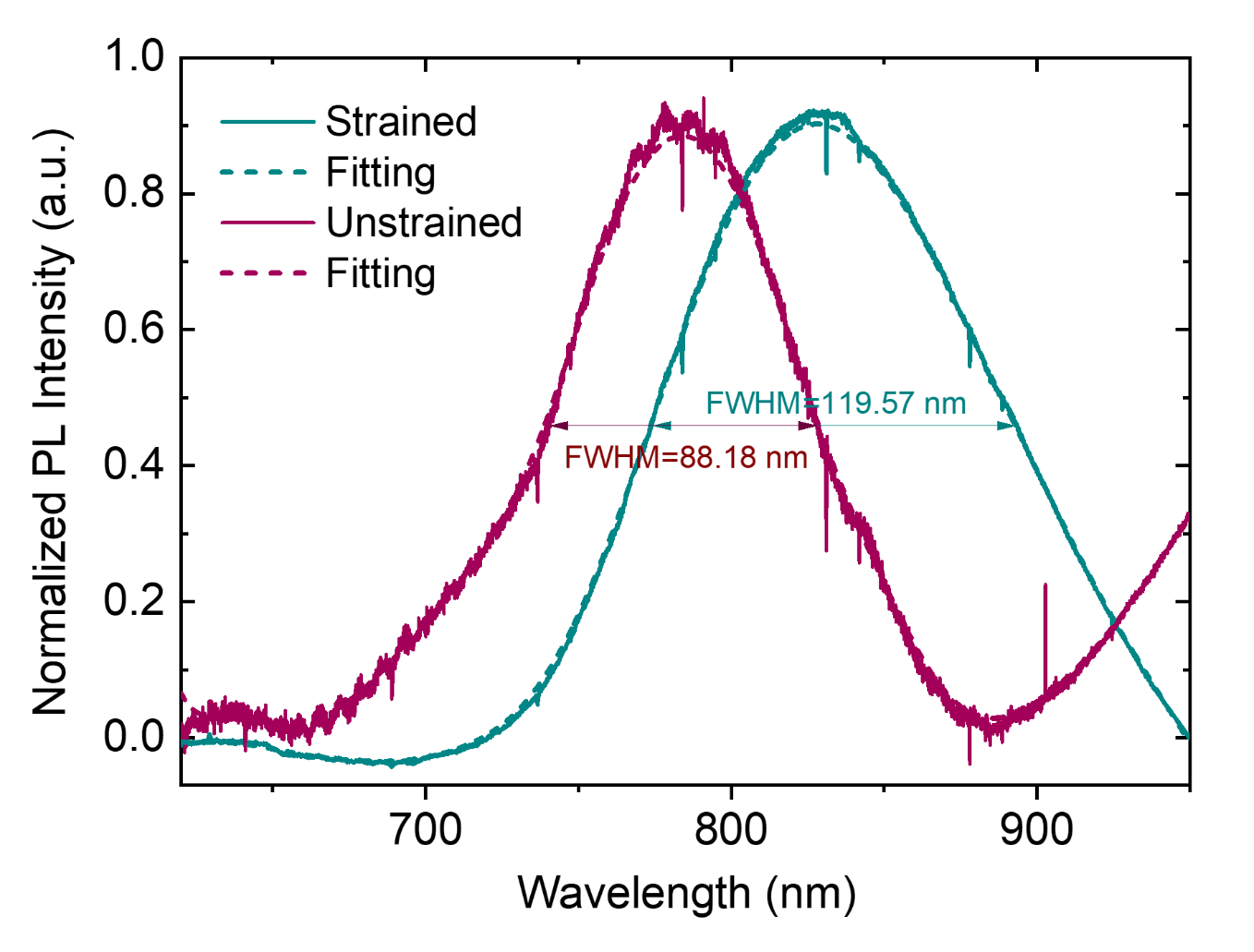


**Fig. S3**. The PL spectra obtained from the unstrained (off-PNRs) and strained hBN (on-PNRs), with fitting performed by a Lorentzian function to extract the full width at half maximum (FWHM). The PL peak-shift in unstrained hBN can be attributed to the base-line fitting.


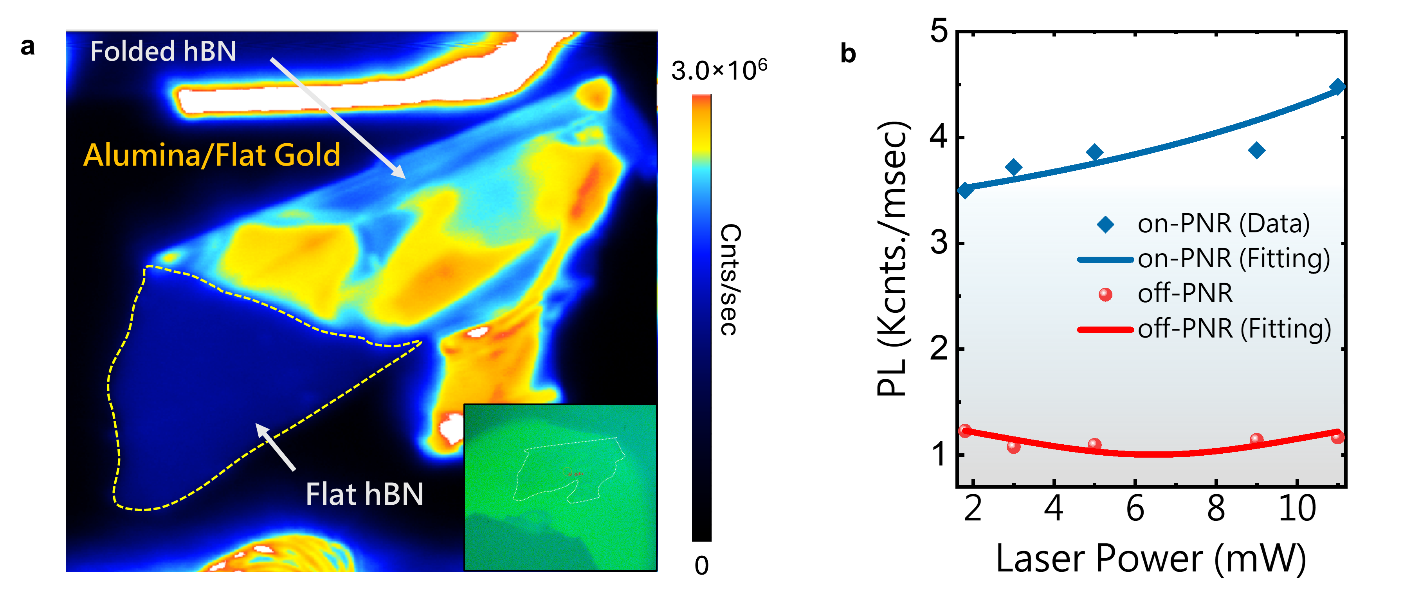


**Fig. S4**. **(a)** Confocal PL map of $V_{B}^{-}$ defects in hBN lying over the flat Al_2_O_3_-coated waveguide, without nanostructures. **(b)** PL intensities of $V_{B}^{-}$ defects on- (blue) and off-PNR (red) as functions of the laser power.


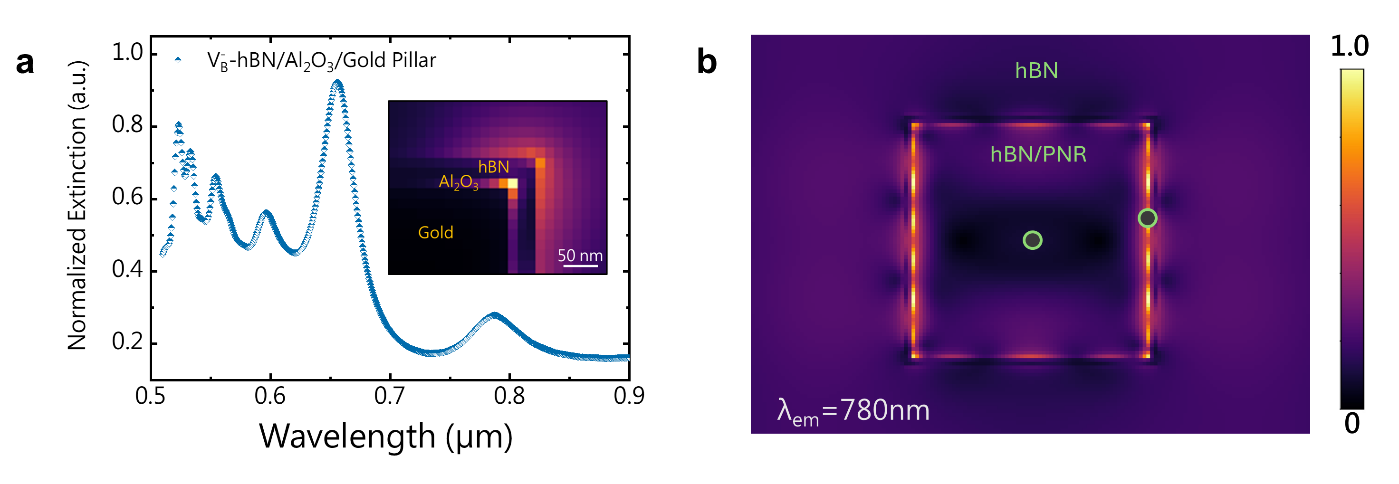


**Fig. S5**. **(a)** The normalized extinction spectrum reveals the wavelength-dependent plasmonic resonance characteristics of PNRs. The inset shows an enlarged image of the e-field concentration at the PNR. **(b)** The edge-driven strain enhancement in $V_{B}^{-}$ PL.


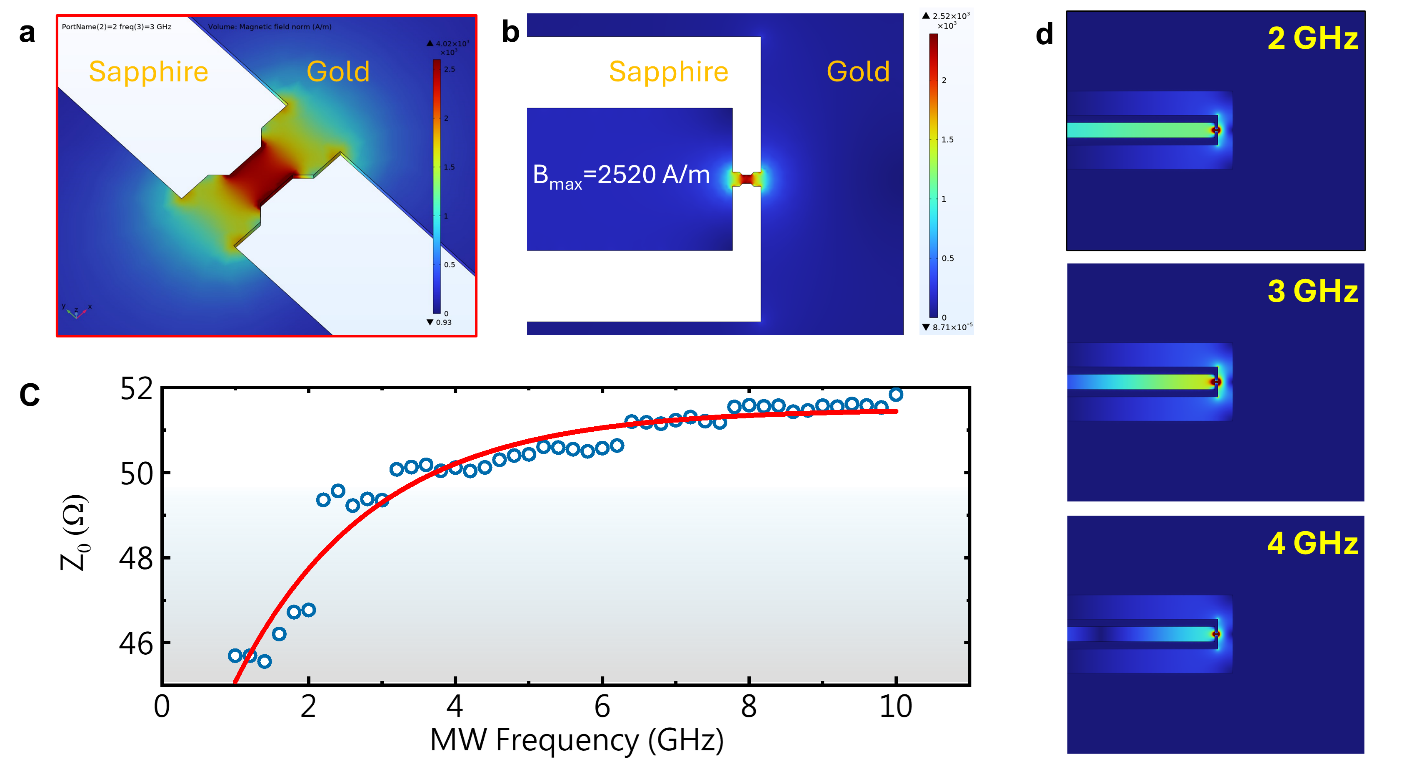


**Fig. S6**. **(a)** COMSOL simulations of a planar coplanar waveguide (CPW) showing field accumulation. (**b**) The quantification of the field accumulation at the constricted part of the CPW using COMSOL simulations. (**c**) The impedance characteristics of CPW as a function of frequency. **(d)** B-field accumulation at the constricted part of the CPW at various MW frequencies (2-4 GHz), showing the optimal results around 3GHz.


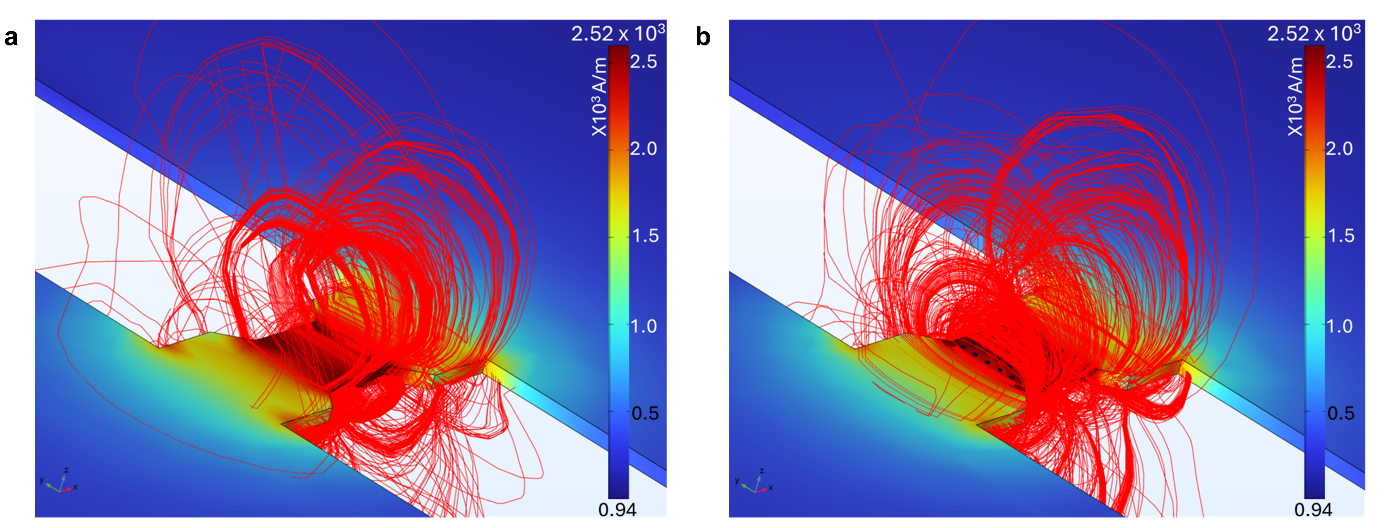


**Fig. S7**. **(a)** COMSOL simulations of a planar coplanar waveguide (CPW) showing field accumulation. (**b**) COMSOL simulations were performed for the nanostructured waveguide using the same parameters, showing almost no effect on field accumulation after introducing PNRs onto the CPW.

**
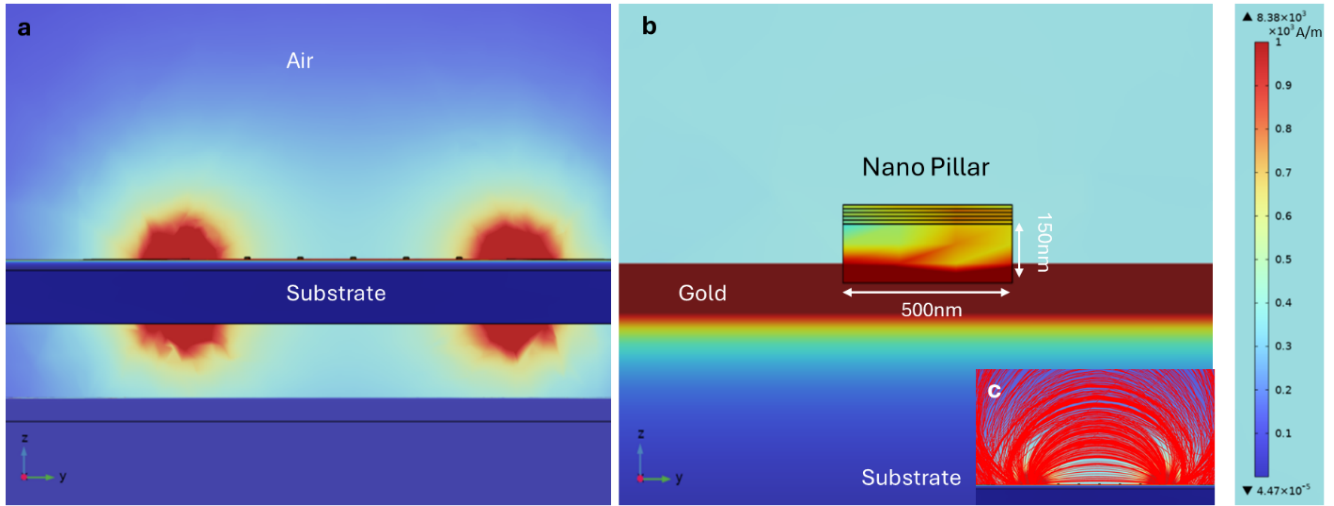
**

**Fig. S8**: (**a**) shows the Magnetic field distribution in the y-z plane of the waveguide. **(b)** shows the zoomed-in figure showing the magnetic field concentration in the region around the nanopillars. Inset c) shows the qualitative visualization of the magnetic field distribution. While the maximum magnetic field is concentrated on the edges, the region near the nano pillars also experiences a significant field.


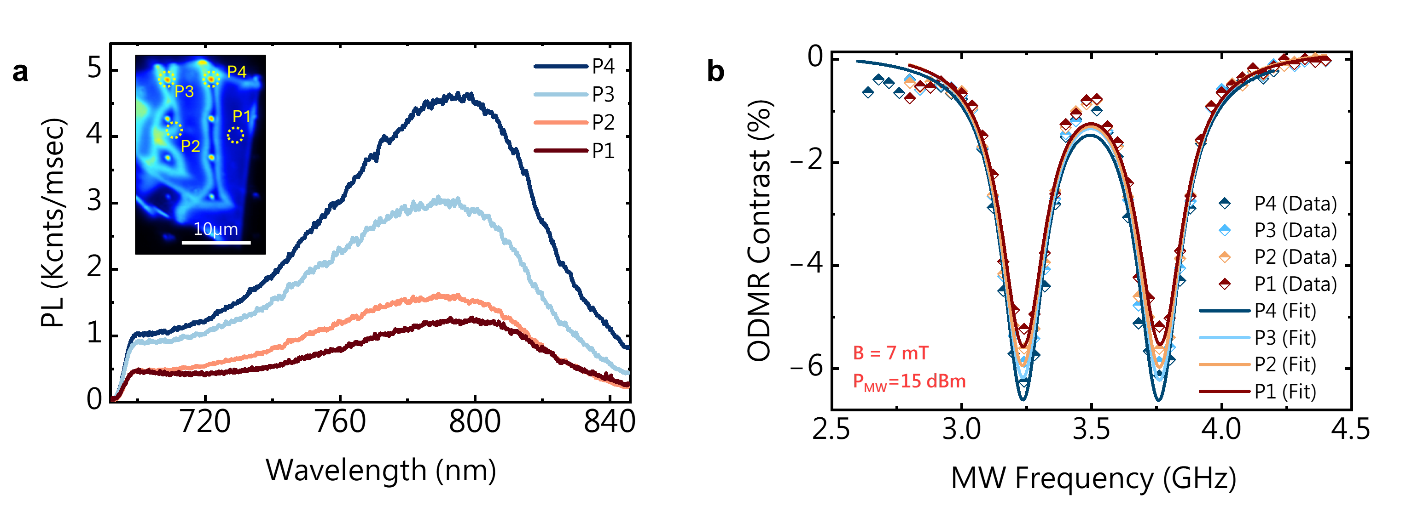


**Fig. S9**. **(a)** Localized PL spectra acquired from points P1-P4, highlighted by yellow circles in the inset. **(b)** *cw* ODMR spectra obtained from points P1-P4, showing strong correlation with the amount of strain. The ODMR spectra were acquired at a MW power of 15 dBm and in the presence of an external magnetic field of 7 *mT*.


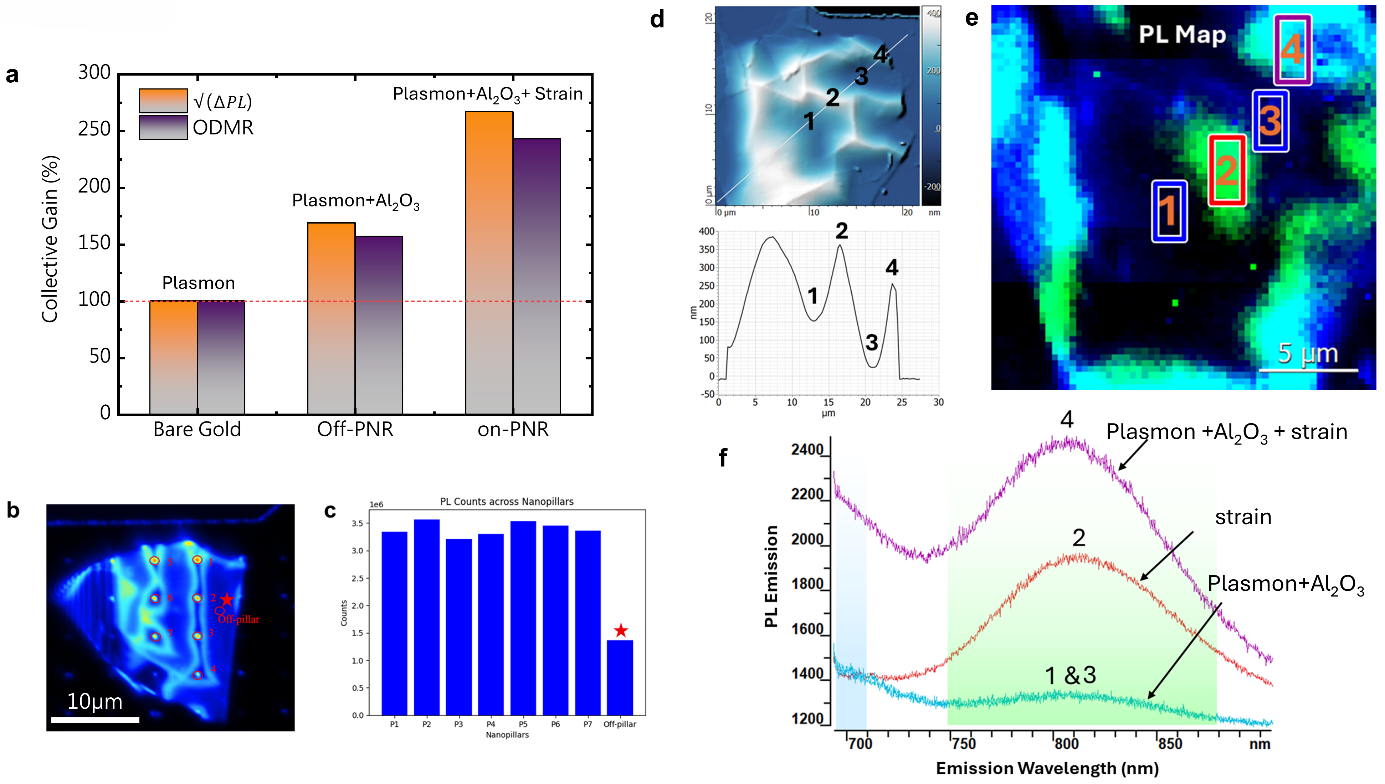


**Fig. S10**. **(a)** Collective gains (%) in √PL and ODMR contrast from pure plasmon, plasmon + Al₂O₃ coating (off-PNR), and subsequent nanopillar integration (on-PNR). **(b)** PL map of the hBN flake. The associated measurement uncertainty is ±1. **(c)** A histogram of PL counts collected over multiple nanopillars shows excellent reproducibility of our device. The associated measurement uncertainty is ±1. **(d)** AFM topography image with corresponding height profile. **(e)** Co-localized PL of *V*_B_⁻ defects from the flake in (d). **(f)** PL spectra acquired at points 1–4 highlighted in (d,e).


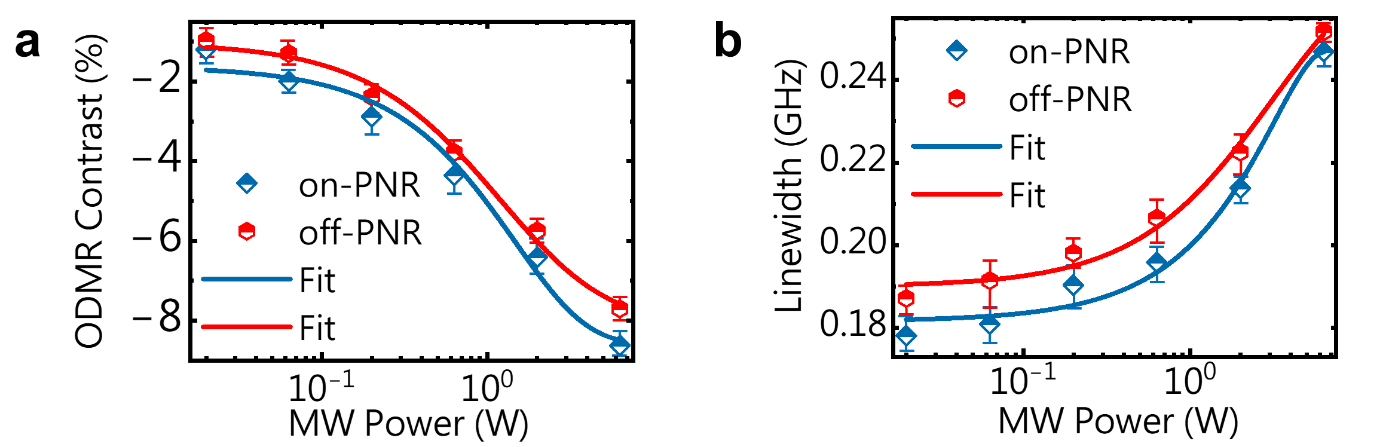


**Fig. S11**. (**a**) MW power dependence of ODMR contrasts for on- and off-PNRs, measured at a magnetic field of 30 mT. (**b**) Variation in the ODMR contrast linewidth (∆υ) from on- and off-PNRs with varying MW power levels to demonstrate power broadening.


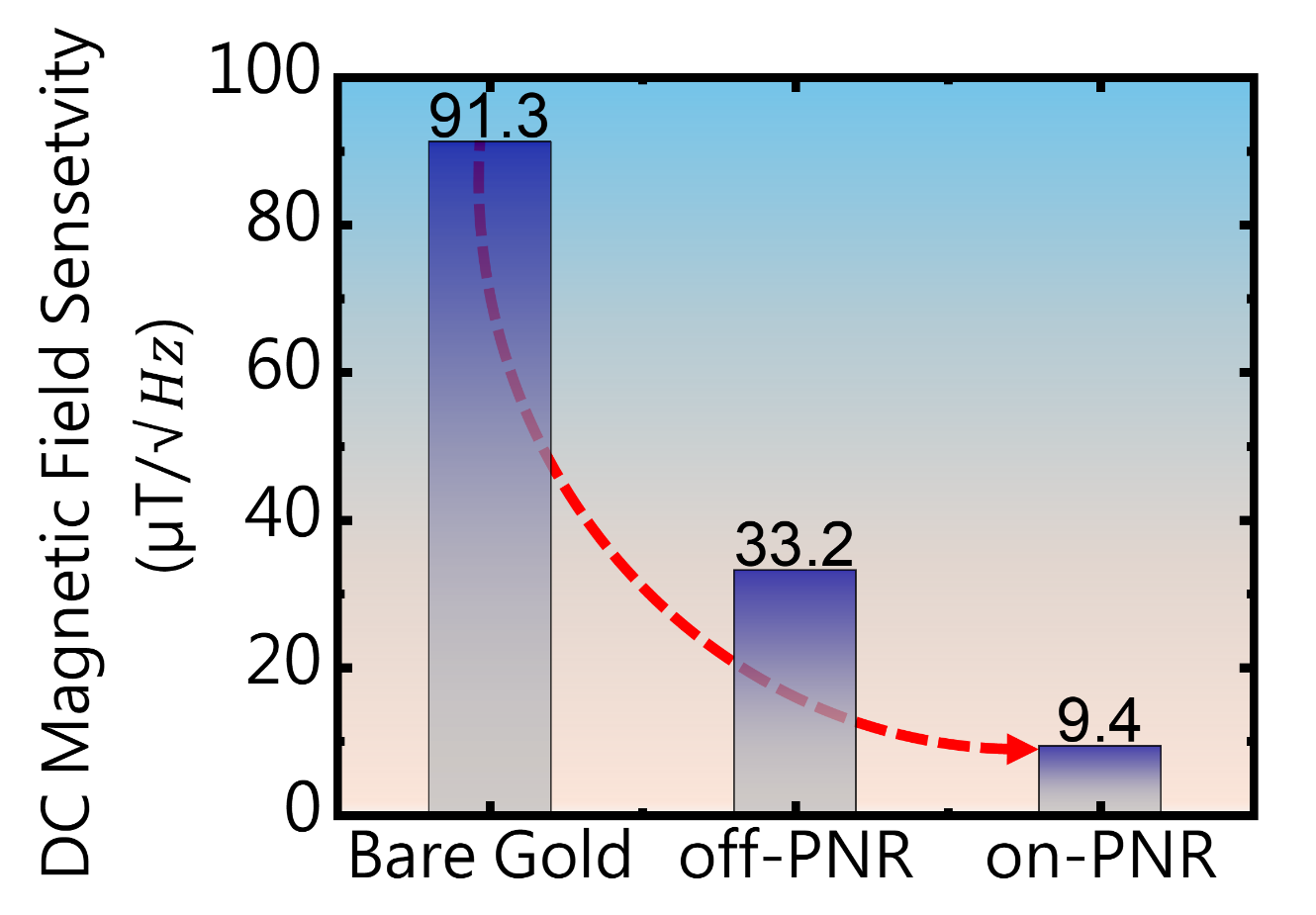


**Fig. S12**. The DC magnetic field sensitivity (*η*_DC_) achieved for $V_{B}^{-}$-hBN lying over bare gold, off-PNR, and on-PNR.


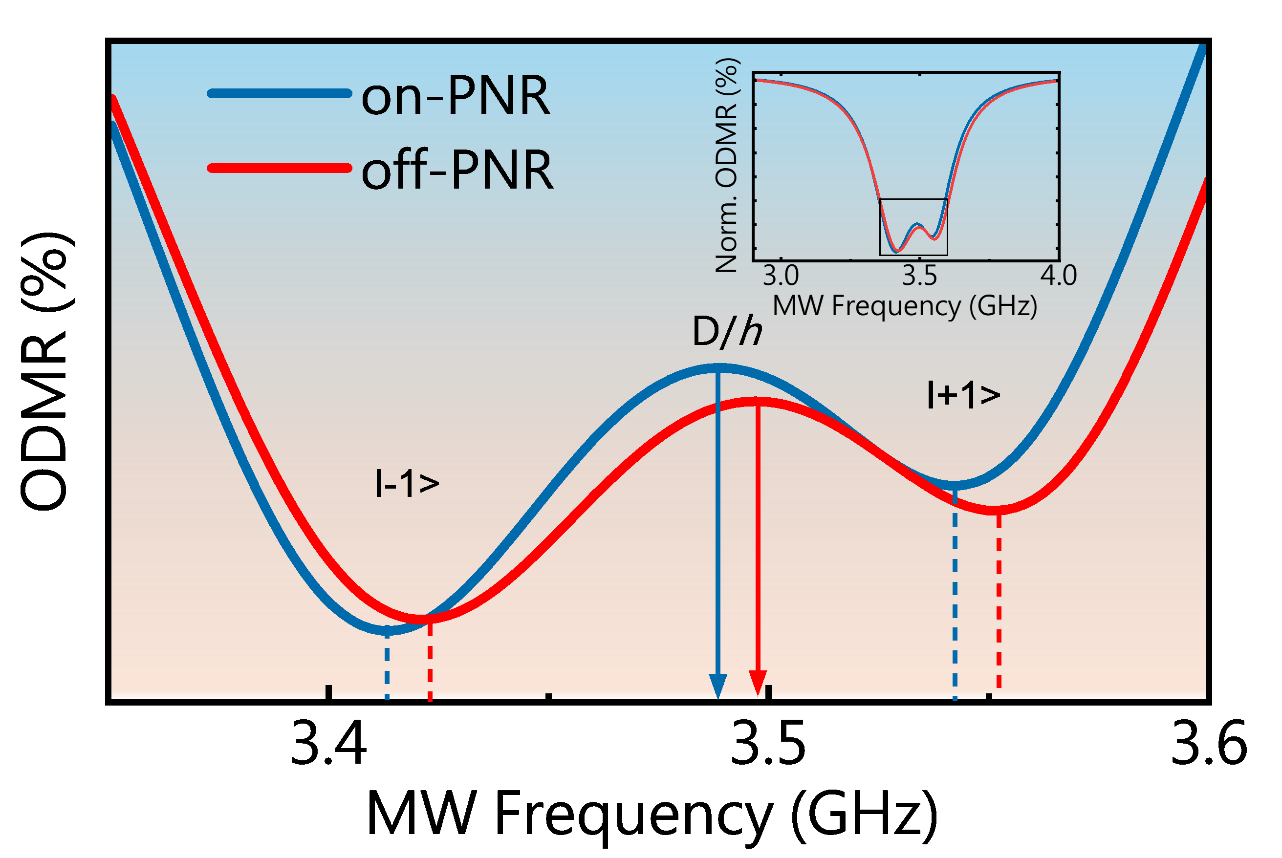


**Fig. S13**. Strain-driven ZFS and spin resonance shift in normalized ODMR spectra, obtained from on- and off-PNR. The inset shows the full spectra.


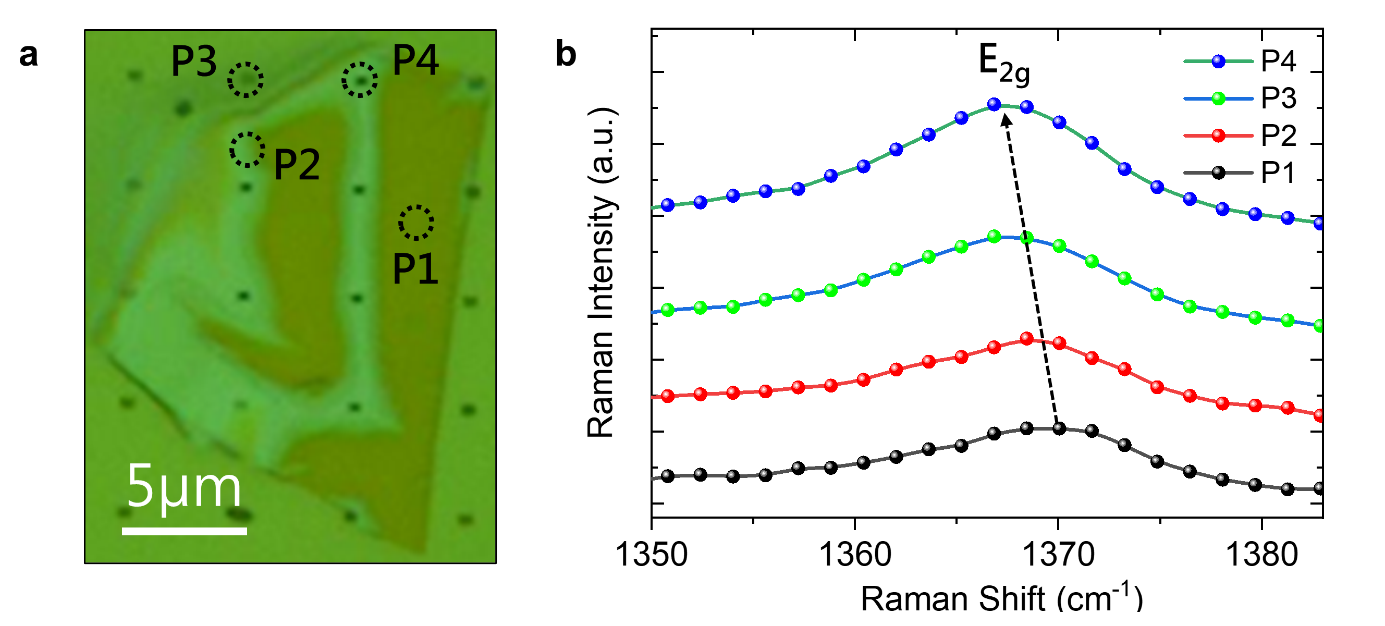


**Fig. S14**. (**a**) The optical micrograph of the hBN-coupled PNRs, showing points P1-P4. (**b**) Corresponding redshift in E_2g_ mode of hBN, showing an increasing tensile strain from P1-P4.


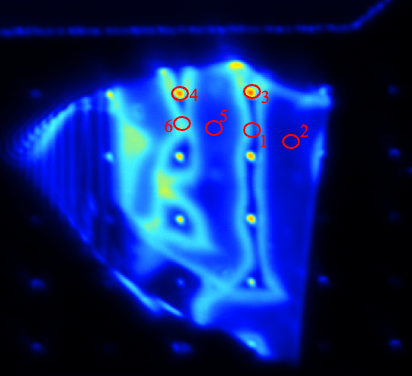

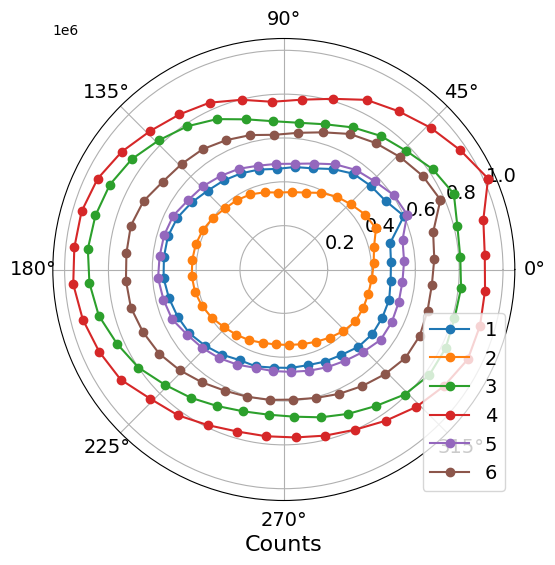


**Fig. S15.** Polarization dependence of the emission from 6 different spots on the sample.
